# The overlooked burden: anti-seizure medications, laxatives, and antipsychotics prescribed in primary care for people with intellectual disability

**DOI:** 10.3389/fpsyt.2026.1714524

**Published:** 2026-02-19

**Authors:** Richard Laugharne, Ian Wilson, Mike Wilcock, Rohit Shankar

**Affiliations:** 1 Adult Intellectual Disability services, Cornwall Partnership NHS Foundation Trust, Truro, United Kingdom; 2 Cornwall Intellectual Disability Equitable Research (CIDER), University of Plymouth Peninsula School of Medicine, Truro, United Kingdom; 3 Cornwall and Isles of Scilly Integrated Care Board, Chy Trevail, Bodmin, United Kingdom; 4 Pharmacy Department, Royal Cornwall Hospitals Trust, Truro, United Kingdom

**Keywords:** antipsychotics, constipation, developmental disorder, epilepsy, seizures

## Abstract

**Objectives:**

People with intellectual disabilities (PwID) have higher prevalences of epilepsy and constipation than the general population. Constipation is having fewer than three bowel movements or requiring laxatives three or more times weekly. Both epilepsy and constipation contribute significantly to premature mortality. To manage constipation, many individuals are prescribed long-term laxatives, which serve as a surrogate indicator of constipation. PwID and epilepsy also have a high prevalence of multimorbidity and polypharmacy, particularly involving antiseizure medications (ASMs) and antipsychotics. This study aimed to explore associations between ASM use, antipsychotics, and laxative prescribing.

**Method:**

The primary care dataset in Cornwall, England (population 572,000), was used to examine patient prescribing records for laxatives, ASMs, and antipsychotics using SNOMED diagnosis codes for PwID. Age and sex were recorded. Results are reported as the prevalence of study cohorts.

**Results:**

Of 3,189 PwID in Cornwall’s GP registers, 2,799 (88%) were over 18, and 1,881 (59%) were men. Among them, 725 (23%) were prescribed laxatives and 467 (15%) ASMs. Of these, 209 were on both laxatives and ASMs (28.8% of all laxative users, 44.8% of all ASM users). Older PwID (> 40 years) were more likely to be on both ASMs and laxatives. Among the 209, 72 (34.4%) were on antipsychotics, with overrepresentation in those over 40.

**Conclusion:**

Nearly half of PwID on ASMs are prescribed laxatives, and over a third of them were also found to be taking antipsychotics. Given the significant links among epilepsy, constipation, and antipsychotic use to premature mortality in PwID, it is imperative to consider these factors collectively rather than individually.

## Background

The prevalence of seizures in people with intellectual disability in England is estimated at 22.2%, compared with 1% in the general population (1, 2). Furthermore, epilepsy in people with intellectual disability is a bellwether for numerous negative health determinants, such as multimorbidity, polypharmacy, treatment-resistant epilepsy, increased emergency department (ED) admissions, and premature mortality (3–7). These outcomes are additionally influenced by age and the nature of care received (3, 8). Older adults with intellectual disability (those over 40 years of age) have significantly worse outcomes, including higher numbers of antiseizure medications (ASMs) despite experiencing fewer seizures (3, 9).

Like epilepsy, constipation too has increasingly been recognised as a significant problem for people with intellectual disability (10). Most prevalence surveys suggest 33%–50% of people with intellectual disability suffer from constipation (10). Constipation has been shown to be more prevalent in people with epilepsy compared to those without, likely due to the adverse effects of both epilepsy and/or its treatment (11). For people with intellectual disability, constipation has been reported to be more prevalent in those with comorbid epilepsy than in those without (12). However, others have described limitations in the current literature on this topic, such as differences in defining the study population and the diagnoses of interest (13).

Notwithstanding these inadequacies in the literature, constipation is recognised as contributing to increased ED admissions and premature mortality (7, 12). Risk factors for constipation in people with intellectual disability include age and side effects of medication, particularly ASMs, opioids, and psychotropic medications (14–16). A recent study found that, of 46 admissions of adults with intellectual disability diagnosed with constipation to two general hospitals in southwest England over a 5-year period, 41% were on an ASM, whilst 5% were on antipsychotic treatment (APT) and opioids (17).

The pharmacological links between APT and ASMs, particularly in relation to constipation, are well recognised (18). However, the clinical intersection of constipation, seizures, ASMs, and other significant medications such as APTs is not well recognised in routine clinical practice, even in the general population (13, 19). This issue is particularly relevant for people with intellectual disabilities and epilepsy. ASMs and antipsychotic medications are independently associated with an increased risk of constipation, yet they are frequently prescribed together for people with intellectual disabilities without adequate consideration of the potential impact on bowel function.

A recent study showed that the prevalence of APT prescribing in people with intellectual disability and epilepsy in secondary care in England and Wales is 27% (4). A follow-up study in secondary care in England and Wales identified APT as a significant risk factor for premature mortality in people with intellectual disability and epilepsy (20).

Most people with intellectual disability are managed in the community in primary care, yet little research has examined bowel health in this population (14). A surrogate consequence of constipation is laxative prescribing (21). Around 5% of people with intellectual disability are prescribed regular laxatives in any 12-month period compared to 0.1% of the general population (22). Similarly, most people with intellectual disability and epilepsy are on ASMs (Branford et al., 2023b).

We sought to understand, in primary care, whether there is an association between the prescribing of ASMs and laxatives in people with intellectual disability. Furthermore, we aimed to identify the prevalence of APT prescribing in people with intellectual disability on ASMs and laxatives.

## Methods

The STROBE guidance for cross-sectional studies was followed to report the study findings (**Supplementary Material 1**).

### Study design

This retrospective cross-sectional study used medicines, health, and demographic data for people with intellectual disability extracted from an anonymised common primary care medical dataset derived from the general practice surgeries across the Cornwall and Isles of Scilly Integrated Care Board. The searches were run on 23 November 2023 using the Systematized Nomenclature of Medicine (SNOMED). SNOMED is a structured clinical vocabulary for use in an electronic health record (23). The primary care database contains data on demographics, diagnoses using the SNOMED classification, prescriptions, and other aspects of patient care. A search for patient records with a SNOMED code indicating intellectual disability and associated terminology (i.e., learning disability/mental handicap/mental retardation) was undertaken (**Supplementary Materials 2**, **3**). The earliest event date corresponds to the earliest diagnosis date.

This cohort dataset was further searched for prescription items identified within the British National Formulary (BNF) by section and subparagraph within the relevant chapters. The BNF is a reference book for healthcare professionals in the UK that provides a wide range of information and guidance on prescribing and pharmacology, as well as specific information about various medications available through the UK NHS (18). The BNF codes are hierarchical. The first characters indicate which part of the BNF a drug belongs to. For example, drugs in BNF Chapter 1 (Gastrointestinal System) always begin with “01”. The BNF is then further subdivided into sections. For example, laxatives, contained within Chapter 1 Section 6 of the BNF, all begin with “0106”. BNF codes do not easily or comprehensively map to the Anatomical Therapeutic Chemical Classification because of differences in the coding systems. The specific classes of medications investigated were laxative prescribing (BNF 01.06), ASM prescribing (BNF 04.08.01), and drugs used for psychoses and related disorders prescribing (BNF 04.02.01).

Algorithms were developed to link SNOMED codes to the presence or absence of the target prescriptions, and Structured Query Language processes were used to run searches. Details of the searches undertaken are provided in **Supplementary Material 4**.

### Procedure overview

The Cornwall and Isles of Scilly Integrated Care Board (CIOSICB) Information Technology (IT) team first identified all people with intellectual disability and then further separated those who were prescribed ASM and laxative medication. Additional queries were created to determine whether individuals were on laxatives but not ASMs, or on ASMs but not laxatives. The data were stratified by age and gender and subsequently used to identify a cohort of people with intellectual disabilities who were prescribed antipsychotic medication.

### Ethics and governance

The project was an internal audit/service evaluation, and data were collected in compliance with the General Data Protection Regulation (GDPR). According to the NHS Health Research Authority tool (http://www.hra-decisiontools.org.uk/research/index.html), no formal ethical approval was required for this study (**Supplementary Material 5**). No patient-identifiable data were collected.

### Analysis

Statistical analysis focused on summarising data from the cohort of patients, with categorical variables summarised by the number and percentage in each category.

## Results

Data on 3,189 patients (59% men, 88% aged over 18 years) with intellectual disability in Cornwall and the Isles of Scilly were extracted from the database. The population of the area is 572,000, representing 0.56% of the population.

**Figure 1:**
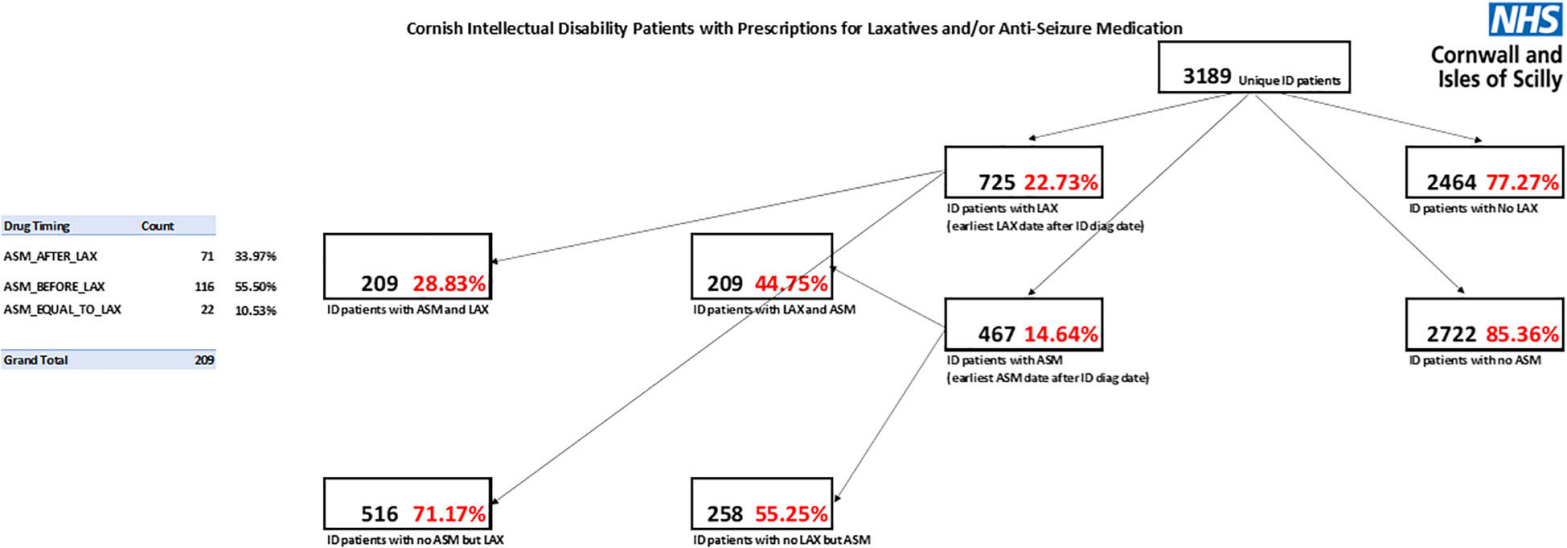
Cornish intellectual disability patients with prescriptions for laxatives and/or antiseizure medication.

Of these 3,189 people, 725 (22.7%) were prescribed laxatives and 467 (14.6%) were prescribed ASMs (**Table 1**; **Figure 1**). Of the 467 prescribed ASMs, 209 (44.8%) were also prescribed laxatives, which is twice the rate of the entire cohort of people with intellectual disability. Of the 725 people on laxatives, the same number, 209, were also on ASMs, representing 28.8% of those on laxatives, similarly twice the rate of the entire cohort of people with intellectual disability.

**Table 1:** People with intellectual disabilities in Cornwall: numbers prescribed antiseizure medication and/or laxatives.

	Laxative yes	Laxative no
ASM yes	209	258
ASM no	516	2,206

Among 3,189 people with intellectual disabilities, laxative use was significantly more common in those prescribed antiseizure medication. A Chi-square test of independence showed a statistically significant association between antiseizure medication use and laxative prescribing (*p* < 0.001). Patients receiving antiseizure medication had approximately 3.5 times higher odds of laxative use compared with those not receiving antiseizure medication.

For the 209 people with intellectual disability on both ASMs and laxatives, 116 (56%) were prescribed ASMs first, followed by laxatives; 71 (34%) received the medications in the reverse order, and for 22 (11%), both medication classes were recorded as commencing simultaneously.

Among those over 18 years of age (*n* = 2,799), 1,445 (men: 893) were aged 19–40, and 1,354 (men: 760) were over 40. Of those over 18, 631 (22.5%) were prescribed laxatives, including 247 (39% men: 127) in the 19- to 40-year-old age group and 344 (61% men: 202) aged 40 or over.

Similarly, 438 of the 2,799 (15.7% men: 246) were on ASMs. There were 188 people with intellectual disability (43% men: 125) between 19 and 40 years old, while 250 (57% men: 121) were 40 years or older. Of the total population of 2,799 adults with intellectual disability, 199 (7%) were on both laxatives and ASMs. This represents 45% of those on ASMs and 31.5% of those on laxatives.

Of the 199 on both ASMs and laxatives, 72 (36% men: 45) were on antipsychotics, of whom 52 (72% men: 30) were over 40 years old.

Details of adults are provided in **Supplementary Material 6**.

## Discussion

We used data on prescribing for 3,189 people with intellectual disability, of whom 390 were children and young people (CYP) and 2,799 adults from one county (population: 572,000) in southwest England. This represented 0.37% of CYP in Cornwall, which is lower than the national average of 2.5% (24, 25). Similarly, for adults, our study sample represented 0.49%, which is lower than the 2.16% expected (24, 25). This finding is consistent with existing literature, where large discrepancies are observed in database recording of intellectual disability as a diagnosis, particularly in childhood, due to a range of established reasons (26). It is worth noting that reliable evidence indicates primary care registers generally record 0.5% prevalence for people with intellectual disability, particularly among adults (27, 28). This is consistent with our study findings.

Nearly one-quarter of individuals were prescribed laxatives, which is consistent with data from previous research (14). Approximately 15% were prescribed ASMs, which is lower than expected, as 22% of people with intellectual disabilities are estimated to have co-occurring epilepsy (1). A large cross-sectional analysis of a primary care database in 2012, covering 408 English general practices and examining the health characteristics of people with intellectual disabilities, reported that 18.5% of the study population had recorded epilepsy (27). More recent evidence shows that ASM prescribing varies by decade of age, ranging from 6% in the first decade to 21.5% by the fifth decade (9). These findings suggest that variations in prescribing may not directly reflect the prevalence of epilepsy.

Addressing our research question, among those on ASMs, just under half were also prescribed laxatives, double the rate of laxative prescribing in the entire people with intellectual disability cohort. Similarly, among those on laxatives, there was twice the rate of ASM prescriptions. These findings suggest an association between ASM and laxative prescribing in people with intellectual disability. A particular concern was the higher number of people with intellectual disability over 40 who were on both ASMs and laxatives. It has been recognised that people with intellectual disability over 40 with epilepsy have higher risks of polypharmacy and multimorbidity (3, 8). It is also concerning to note that, among those adults prescribed laxatives and ASMs, over a third were likely to be on an antipsychotic as well. The concern increases when age is considered. Nearly three of four people on antipsychotics, laxatives, and ASMs were over 40. Although these numbers are small, they suggest the presence of a high-risk group that becomes increasingly vulnerable, particularly after 40 years of age, and is subject to various negative determinants of health, including premature mortality (20). These findings are supported by other recent studies (4, 29).

### Limitations

Specific and detailed cohort characteristics are unavailable due to the nature of the study.

Epilepsy is a risk factor for constipation and is also associated with irritable bowel syndrome (IBS). IBS is more prevalent in autistic people, and many people with intellectual disability are also autistic. Overflow diarrhoea is another bowel issue that receives little attention in the literature for people with intellectual disability and, in some cases, can be a symptom of constipation. Furthermore, some ASMs can cause diarrhoea as a side effect. Some ASMs are used as mood stabilisers, but evidence suggests considerable overlap (30). We cannot comment on the purpose of the prescribed antipsychotic or antiseizure medication, as the purposes of prescribing both medications are multiple. We did not distinguish between first- or second-generation antipsychotics or among different antiseizure medications. Furthermore, we could not expect the side effects of ASMs to differ depending on the indication. These complex confounders have not been considered in this study.

As discussed above, the prevalence of ID and ASM prescribing in people with intellectual disability is lower than expected but consistent with primary care research. Only data coded in the general practice electronic health record and thus identified through the dataset were included in the analysis. Any free-text data were not included; therefore, our analysis may underreport parameters entered as free text rather than coded. Data in this dataset are collected principally for the administration of the primary care practice and may not be recorded with the timeliness and precision associated with a prospective register study. Others have also reported issues with GP recording of the prevalence of people with intellectual disability (28). Our analysis does not account for over-the-counter laxative use, as this would not typically be recorded in the health record. Consequently, the actual rate of laxative use may be higher than reported. Our dataset arises from one geographical region in the southwest and may not be generalisable.

One of our key findings is the association between ASMs, laxatives, and antipsychotics, particularly in those over 40. Cornwall is an outlier, as there is active research and clinical focus on psychotropic reduction across the system (19, 31–34). Consequently, these associations might be weaker than in other geographical areas. Furthermore, we have not undertaken any tests of significance between subgroups, as this was an exploratory study aimed at enumerating associations.

## Conclusion

This study suggests an association between the prescription of ASMs and laxatives. Furthermore, there is an association between prescribing antipsychotics when both laxatives and ASMs are used. The polypharmacy trend appears to increase with age, being highest for those over 40 years. This is the age at which many people with intellectual disability and epilepsy may have moved to care settings, developed other health conditions, and face an increasing risk of premature mortality. Increased vigilance is needed to actively and regularly manage constipation and epilepsy through routine medication reviews of laxatives and ASMs. This is particularly needed for individuals over 40 and/or prescribed antipsychotics. Active recognition of this vulnerable subcohort could allow them to benefit from more informed and personalised annual health checks, potentially saving lives (35, 36).

This study is an explorative analysis of key associations and presents data from a primary care cohort, adding evidence often obtained only from secondary care cohorts. The cohort size in this study is larger than that in many other studies. The study reveals associations rather than causations. Unfortunately, other demographic and health characteristics, whose analysis could likely strengthen the findings, were not available. It can be hypothesised that either epilepsy or the prescribing of ASMs may increase the risk of constipation, but this requires further research.

## Data Availability

The original contributions presented in the study are included in the article/**Supplementary Material**. Further inquiries can be directed to the corresponding author.
