# Supplementary material for: The overlooked burden: anti-seizure medications, laxatives, and antipsychotics prescribed in primary care for people with intellectual disability
Source: Front Psychiatry. 2026 Feb 19;17:1714524. doi: 10.3389/fpsyt.2026.1714524 (PMC12960475; doi:10.3389/fpsyt.2026.1714524)
Supplement: Supplementary file 2 [file Table2.docx]

**Search Strategy:**

**Procedure:** Structured Query Language (SQL) process to find people diagnosed with Intellectual Disability (ID) who were then prescribed either Anti Seizure Medication (ASM), Anti Psychotic Medication (APT) and/or Laxatives.

**Date:** 23/11/2023

**Procedure overview**

The Cornwall and Isles of Scilly Integrated Care Board (CIOSICB) IT team to identify Intellectual Disability (ID) people who were then prescribed anti seizure medication (ASM) and laxative medication. Two further requests were made to split this data by age and gender , and then to identify a cohort of ID people who were prescribed anti psychotic medicine (APT). Further queries were created to establish if people were on , for example, laxatives but not ASM, or laxatives, ASM and APT medication.

**Requirements**

1. Identify ID people from the CIOSICB’s primary care dataset.
2. Identify ID people prescribed laxatives.
3. Identify ID people prescribed ASM medication.
4. Identify ID people prescribed both laxatives and ASM medication.
5. Identify which drug was prescribed first out of the above cohort.
6. Identify ID people prescribed laxatives but not ASM medication.
7. Identify ID people prescribed ASM medication but not laxatives.
8. Split the above cohorts by gender and age – groupings 19-40 and 40 + years of age.
9. Identify ID people prescribed with APT medication.
10. Identify ID people prescribed all of laxatives, ASM and APT medication.
11. Identify which drug was prescribed first out of APT and laxatives and APT and ASM.
12. Identify ID people prescribed laxatives and APT but not ASM medication.
13. Identify ID people prescribed ASM and APT medication but not laxatives.
14. Identify ID people prescribed APT medication but not laxatives nor ASM.
15. **Identify ID People**

The first element of this project was to create a cohort of people that had a diagnosis of Intellectual Disability (ID). The CIOSICB primary care dataset was searched for people with a snomed code description of ‘%Intellectual Disability%’. Further to this, the dataset has identified people with a snomed code that matches a code used for the QOF disease register. Codes are available in the appendix. This query grouped the output by gender and age group , then a list of unique individuals from this cohort was generated , along with the earliest event date , translated into the earliest diagnosis date (as one person may have had a matching snomed code event on several occasions.)

1. **Identify ID People prescribed Laxatives**

The CIOSICB primary care dataset also contains a prescribed medication table. From this, any prescribed medication that matched the BNF code of ‘0106%’ was extracted , then a unique list of people with their earliest laxative prescription date was created.

1. **Identify People who are in both the ID and Laxative datasets**

From the two tables, we can join them together using the person’s individual unique identifier, and add a filter that the laxative earliest medication start date has to be after the person’s earliest diagnosis date.

SELECT *

FROM #LD_PATIENTS

JOIN #LAX_PATIENTS ON (#LD_PATIENTS.EVENT_SKEY_PATIENT = #LAX_PATIENTS.LAX_MEDICATION_SKEY_PATIENT

AND #LAX_PATIENTS.LAX_EARLIEST_MEDICATION_START_DATE >= #LD_PATIENTS.EARLIEST_EVENT_DIAGNOSIS_DATE)

1. **Identify People who are in the ID dataset but not Laxative dataset**

Making the change from inner to left outer join, and filtering where the laxative unique identifier is null means we can find those people with ID but no laxative prescription.

SELECT *

FROM #LD_PATIENTS

LEFT OUTER JOIN #LAX_PATIENTS ON (#LD_PATIENTS.EVENT_SKEY_PATIENT = #LAX_PATIENTS.LAX_MEDICATION_SKEY_PATIENT

AND #LAX_PATIENTS.LAX_EARLIEST_MEDICATION_START_DATE >= #LD_PATIENTS.EARLIEST_EVENT_DIAGNOSIS_DATE)

WHERE LAX_MEDICATION_SKEY_PATIENT IS NULL

1. **Identify ID People prescribed Anti-Seizure Medication**

From the prescribed medication table, any prescribed medication that matched the BNF code of ‘%040801%’ was extracted , then a unique list of people with their earliest ASM prescription date was created.

1. **Identify People who are in both the ID and ASM datasets**

From the two tables, we can join them together using the person’s individual unique identifier, and add a filter that the ASM earliest medication start date has to be after the person’s earliest diagnosis date.

SELECT *

FROM #LD_PATIENTS

JOIN #ASM_PATIENTS ON (#LD_PATIENTS.EVENT_SKEY_PATIENT = #ASM_PATIENTS.ASM_MEDICATION_SKEY_PATIENT

AND #ASM_PATIENTS.ASM_EARLIEST_MEDICATION_START_DATE >= #LD_PATIENTS.EARLIEST_EVENT_DIAGNOSIS_DATE)

1. **Identify People who are in the ID dataset but not ASM dataset**

Making the change from inner to left outer join, and filtering where the ASM unique identifier is null means we can find those people with ID but no ASM prescription.

SELECT *

FROM #LD_PATIENTS

LEFT OUTER JOIN #ASM_PATIENTS ON (#LD_PATIENTS.EVENT_SKEY_PATIENT = #ASM_PATIENTS.ASM_MEDICATION_SKEY_PATIENT

AND #ASM_PATIENTS.ASM_EARLIEST_MEDICATION_START_DATE >= #LD_PATIENTS.EARLIEST_EVENT_DIAGNOSIS_DATE)

WHERE ASM_MEDICATION_SKEY_PATIENT IS NULL

1. **Identify ID People who are in both Laxative and ASM Cohorts and display which prescription occurred first**

Amending the flow of the previous tables provides the people that appear in both datasets. Once the temporary table of ID people was created, another temporary table that included those ID people on laxatives using the previous logic was formed. From there, the temporary table of ASM people was created and an inner join on the two tables to match those ID and laxative people with the ASM dataset. A case when (highlighted yellow) was added to distinguish between which prescription came first.

SELECT t1.SKEY_PATIENT AS LAX_MEDICATION_SKEY_PATIENT,

MIN(t1.MEDICATIONSTARTDATE) AS LAX_EARLIEST_MEDICATION_START_DATE

INTO #LAX_PATIENTS

from

(SELECT DISTINCT c.SKEY_PATIENT, a.MEDICATIONSTARTDATE, a.MEDICATIONENDDATE

,a.BNFCODE,a.RUBRIC

,a.MEDICATIONQUANTITY, a.MEDICATIONQUANTITYUNITS

,a.MEDICATIONDOSAGE,a.PRESCRIPTIONTYPE,a.DRUGSTATUS,a.SOURCESYSTEM

FROM dbo.F_C_GPD_MEDICATION a

JOIN dbo.D_C_GPD_PATIENT b ON b.SKEY_GPD_PATIENT = a.SKEY_GPD_PATIENT

JOIN dbo.D_C_MPI_PATIENT c ON c.SKEY_PATIENT = b.SKEY_PATIENT

WHERE BNFCODE LIKE '0106%'

AND c.SKEY_PATIENT > 0

)t1

GROUP BY t1.SKEY_PATIENT

SELECT *

INTO #LDANDLAX_PATIENTS

FROM #LD_PATIENTS

JOIN #LAX_PATIENTS ON (#LD_PATIENTS.EVENT_SKEY_PATIENT = #LAX_PATIENTS.LAX_MEDICATION_SKEY_PATIENT

AND #LAX_PATIENTS.LAX_EARLIEST_MEDICATION_START_DATE >= #LD_PATIENTS.EARLIEST_EVENT_DIAGNOSIS_DATE)

SELECT t1.SKEY_PATIENT AS ASM_MEDICATION_SKEY_PATIENT,

MIN(t1.MEDICATIONSTARTDATE) AS ASM_EARLIEST_MEDICATION_START_DATE

INTO #ASM_PATIENTS

from

(SELECT DISTINCT c.SKEY_PATIENT, a.MEDICATIONSTARTDATE, a.MEDICATIONENDDATE

,a.BNFCODE,a.RUBRIC

,a.MEDICATIONQUANTITY, a.MEDICATIONQUANTITYUNITS

,a.MEDICATIONDOSAGE,a.PRESCRIPTIONTYPE,a.DRUGSTATUS,a.SOURCESYSTEM

FROM dbo.F_C_GPD_MEDICATION a

JOIN dbo.D_C_GPD_PATIENT b ON b.SKEY_GPD_PATIENT = a.SKEY_GPD_PATIENT

JOIN dbo.D_C_MPI_PATIENT c ON c.SKEY_PATIENT = b.SKEY_PATIENT

WHERE BNFCODE LIKE '%040801%'

AND c.SKEY_PATIENT > 0

)t1

GROUP BY t1.SKEY_PATIENT

SELECT DISTINCT EVENT_SKEY_PATIENT, EARLIEST_EVENT_DIAGNOSIS_DATE, LAX_EARLIEST_MEDICATION_START_DATE

,ASM_EARLIEST_MEDICATION_START_DATE,GENDER, AGE_GROUP

,CASE WHEN #ASM_PATIENTS.ASM_EARLIEST_MEDICATION_START_DATE>#LDANDLAX_PATIENTS.LAX_EARLIEST_MEDICATION_START_DATE THEN 'ASM_AFTER_LAX'

WHEN #ASM_PATIENTS.ASM_EARLIEST_MEDICATION_START_DATE<#LDANDLAX_PATIENTS.LAX_EARLIEST_MEDICATION_START_DATE THEN 'ASM_BEFORE_LAX'

WHEN #ASM_PATIENTS.ASM_EARLIEST_MEDICATION_START_DATE=#LDANDLAX_PATIENTS.LAX_EARLIEST_MEDICATION_START_DATE THEN 'ASM_EQUAL_TO_LAX' END AS DRUG_TIMING

FROM #LDANDLAX_PATIENTS

JOIN #ASM_PATIENTS ON (#LDANDLAX_PATIENTS.EVENT_SKEY_PATIENT = #ASM_PATIENTS.ASM_MEDICATION_SKEY_PATIENT

AND #ASM_PATIENTS.ASM_EARLIEST_MEDICATION_START_DATE >= #LDANDLAX_PATIENTS.EARLIEST_EVENT_DIAGNOSIS_DATE)

1. **Identify ID People with Laxatives but not ASM**

A change to the joins and temporary tables above ensured people on laxatives but not ASM could be highlighted. Specifically, by creating a temporary table of ID people without ASM use (as per section 7) and joining this to a temporary table of people with laxative use.

SELECT t1.SKEY_PATIENT AS ASM_MEDICATION_SKEY_PATIENT,

MIN(t1.MEDICATIONSTARTDATE) AS ASM_EARLIEST_MEDICATION_START_DATE

INTO #ASM_PATIENTS

from

(SELECT DISTINCT c.SKEY_PATIENT, a.MEDICATIONSTARTDATE, a.MEDICATIONENDDATE

,a.BNFCODE,a.RUBRIC

,a.MEDICATIONQUANTITY, a.MEDICATIONQUANTITYUNITS

,a.MEDICATIONDOSAGE,a.PRESCRIPTIONTYPE,a.DRUGSTATUS,a.SOURCESYSTEM

FROM dbo.F_C_GPD_MEDICATION a

JOIN dbo.D_C_GPD_PATIENT b ON b.SKEY_GPD_PATIENT = a.SKEY_GPD_PATIENT

JOIN dbo.D_C_MPI_PATIENT c ON c.SKEY_PATIENT = b.SKEY_PATIENT

WHERE BNFCODE LIKE '%040801%'

AND c.SKEY_PATIENT > 0

)t1

GROUP BY t1.SKEY_PATIENT

SELECT *

INTO #LDANDNOASM_PATIENTS

FROM #LD_PATIENTS

LEFT OUTER JOIN #ASM_PATIENTS ON (#LD_PATIENTS.EVENT_SKEY_PATIENT = #ASM_PATIENTS.ASM_MEDICATION_SKEY_PATIENT

AND #ASM_PATIENTS.ASM_EARLIEST_MEDICATION_START_DATE >= #LD_PATIENTS.EARLIEST_EVENT_DIAGNOSIS_DATE)

WHERE ASM_MEDICATION_SKEY_PATIENT IS NULL

SELECT t1.SKEY_PATIENT AS LAX_MEDICATION_SKEY_PATIENT,

MIN(t1.MEDICATIONSTARTDATE) AS LAX_EARLIEST_MEDICATION_START_DATE

INTO #LAX_PATIENTS

from

(SELECT DISTINCT c.SKEY_PATIENT, a.MEDICATIONSTARTDATE, a.MEDICATIONENDDATE

,a.BNFCODE,a.RUBRIC

,a.MEDICATIONQUANTITY, a.MEDICATIONQUANTITYUNITS

,a.MEDICATIONDOSAGE,a.PRESCRIPTIONTYPE,a.DRUGSTATUS,a.SOURCESYSTEM

FROM dbo.F_C_GPD_MEDICATION a

JOIN dbo.D_C_GPD_PATIENT b ON b.SKEY_GPD_PATIENT = a.SKEY_GPD_PATIENT

JOIN dbo.D_C_MPI_PATIENT c ON c.SKEY_PATIENT = b.SKEY_PATIENT

WHERE BNFCODE LIKE '0106%'

AND c.SKEY_PATIENT > 0

)t1

GROUP BY t1.SKEY_PATIENT

SELECT DISTINCT EVENT_SKEY_PATIENT, EARLIEST_EVENT_DIAGNOSIS_DATE, #LDANDNOASM_PATIENTS.ASM_EARLIEST_MEDICATION_START_DATE

,#LAX_PATIENTS.LAX_EARLIEST_MEDICATION_START_DATE,GENDER, AGE_GROUP

FROM #LDANDNOASM_PATIENTS

JOIN #LAX_PATIENTS ON (#LDANDNOASM_PATIENTS.EVENT_SKEY_PATIENT = #LAX_PATIENTS.LAX_MEDICATION_SKEY_PATIENT

AND #LAX_PATIENTS.LAX_EARLIEST_MEDICATION_START_DATE >= #LDANDNOASM_PATIENTS.EARLIEST_EVENT_DIAGNOSIS_DATE)

1. **Identify ID People with ASM but not laxatives**

The same code as in section 9 was used, amending the code to remove laxatives and keeping the ASM patients.

1. **Identify ID people prescribed Anti Psychotic Medicine**

The same methodology was used to highlight this cohort as in sections 2 and 5 – where a BNF code of ‘%040201%’ was filtered on instead.

1. **Identify ID people prescribed laxatives, ASM and APT medication**

Using the temporary table methods above, it was possible to highlight the above cohort. By identifying those people in section 8 and then linking on the people that also had been prescribed APT medication. Again a case when was added to discover which prescription occurred first, e.g.

,CASE WHEN #APT_PATIENTS.APT_EARLIEST_MEDICATION_START_DATE>#LDLAXASM_PATIENTS.LAX_EARLIEST_MEDICATION_START_DATE THEN 'APT_AFTER_LAX'

WHEN #APT_PATIENTS.APT_EARLIEST_MEDICATION_START_DATE<#LDLAXASM_PATIENTS.LAX_EARLIEST_MEDICATION_START_DATE THEN 'APT_BEFORE_LAX'

WHEN #APT_PATIENTS.APT_EARLIEST_MEDICATION_START_DATE=#LDLAXASM_PATIENTS.LAX_EARLIEST_MEDICATION_START_DATE THEN 'APT_EQUAL_TO_LAX' END AS APT_DRUG_TIMING_LAX

,CASE WHEN #APT_PATIENTS.APT_EARLIEST_MEDICATION_START_DATE>#LDLAXASM_PATIENTS.ASM_EARLIEST_MEDICATION_START_DATE THEN 'APT_AFTER_ASM'

WHEN #APT_PATIENTS.APT_EARLIEST_MEDICATION_START_DATE<#LDLAXASM_PATIENTS.ASM_EARLIEST_MEDICATION_START_DATE THEN 'APT_BEFORE_ASM'

WHEN #APT_PATIENTS.APT_EARLIEST_MEDICATION_START_DATE=#LDLAXASM_PATIENTS.ASM_EARLIEST_MEDICATION_START_DATE THEN 'APT_EQUAL_TO_ASM' END AS APT_DRUG_TIMING_ASM

1. **Identify ID people with either APT and Laxatives, or APT and ASM**

Again by amending the code to exclude either laxatives or ASM, it was possible to highlight those ID people on APT and laxatives, or APT and ASM, and discover which prescription came first.

1. **Identify ID people with an APT prescription but not Laxatives nor ASM.**

By using the logic above, a temporary table of all those ID people with no laxative use and another with no ASM use was created, these were combined using a full outer join to give all patients, then joined to a temporary table of APT prescribed ID people, to ascertain those with an APT prescription but not laxatives or ASM.
