# Supplementary figures and images for: The overlooked burden: anti-seizure medications, laxatives, and antipsychotics prescribed in primary care for people with intellectual disability
Source: Front Psychiatry. 2026 Feb 19;17:1714524. doi: 10.3389/fpsyt.2026.1714524 (PMC12960475; doi:10.3389/fpsyt.2026.1714524)

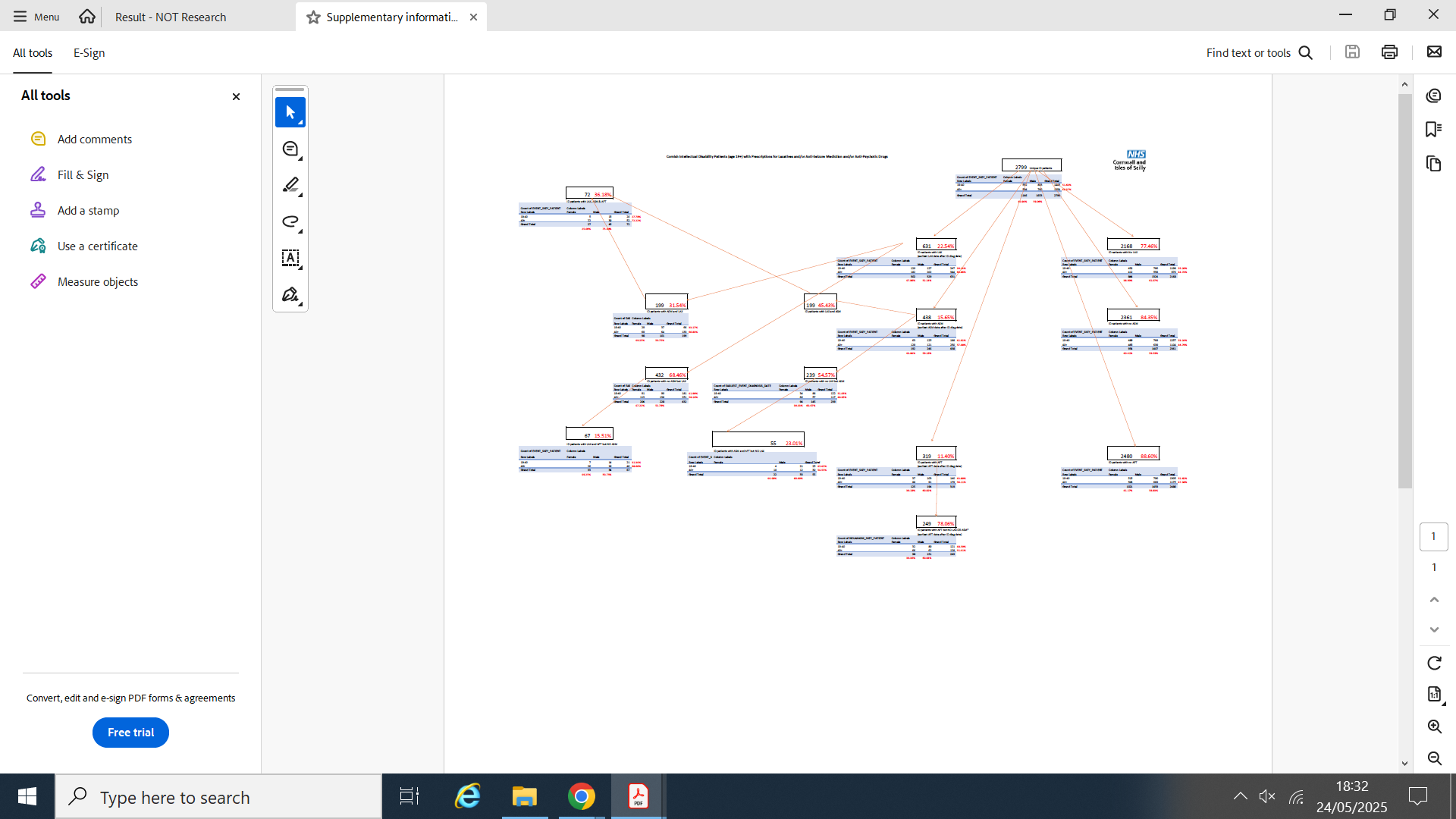

Supplement: Supplementary file 3 [file Table3.docx]
