# Supplementary material for: The overlooked burden: anti-seizure medications, laxatives, and antipsychotics prescribed in primary care for people with intellectual disability
Source: Front Psychiatry. 2026 Feb 19;17:1714524. doi: 10.3389/fpsyt.2026.1714524 (PMC12960475; doi:10.3389/fpsyt.2026.1714524)
Supplement: Supplementary file 4 [file DataSheet1.pdf]

SKEY_SNOMED_CONCEPT	CONCEPT_ID
406673	703389002
487339	719161008
487450	719811001
493071	719016007
501966	716709002
505330	715342005
505360	719826004
505364	718914002
505987	718911005
507703	718912003
507864	718900002
511624	718845002
511891	733090001
512012	718905007
513423	719137001
515121	719138006
515273	732246009
515402	724178000
516369	726709001
516835	726732002
516965	733469003
517127	723441001
517189	719825000
517190	719157002
517630	722213009
518500	734017008
518533	733419006
518595	718909001
519107	718908009
519109	719013004
520500	718896000
520753	723621000
521667	719160009
521691	719018008
522572	719009006
522806	733117001
522967	718897009
523124	732961003
523572	723336008
523896	732251003
524831	725906006
525480	723365002
528334	719010001
528464	732954002
528752	719810000
528817	719012009
528887	725912001
529366	720982007
529883	733455003

	530105	719155005
	531318	733522005
	531833	718846001
	532707	719017003
	533100	733088002
	533131	723304001
	533317	733097003
	533357	719809005
	534492	718910006
	534493	719812008
	535263	723676007
	541286	719842006
	542300	719136005
	542720	733062000
	543451	719156006
	545172	724001005
	547062	720639008
	547315	723994004
	547706	725163002
	548535	726727003
	549281	721089006
	549767	719011002
	554613	719450007
	555523	721087008
	555984	722003007
	565810	719256004
	569050	720981000
	569412	721007005
	578578	722110003
	592716	716107009
	594527	720468000
	609724	720502000
	626245	722478008
	627037	722282008
	653246	718766002
	653447	722456001
	670664	721146009
	673385	722454003
	682805	722379001
	683101	236529001
	684185	722111004
	688442	720501007
	692367	720517001
	696322	719042007
	696750	721973006
	714125	721841001
	725473	720979002
	727004	722209002
	742223	722455002
	746922	720980004

	771533	716706009
	775973	717222003
	791864	722459008
	813317	719396000
	815590	721207002
	821193	720987001
	839816	763598005
	842892	764959000
	845015	763861000
	848099	763744009
	850995	764861005
	853718	763741001
	853938	763743003
	855826	412787009
	856243	763404001
	857197	422437002
	857389	766824003
	857937	765471005
	857972	734349003
	858542	763745005
	858545	765434008
	859007	765761009
	861936	763344007
	866528	763665007
	867489	763625008
	868290	763615003
	869625	764950001
	869649	763136000
	870780	33982008
	871372	763626009
	872837	6.2961E+13
	873569	765089003
	877228	763742008
	878229	763350002
	879019	768677000
	891379	6.3291E+13
	1056765	1156584007
	1051667	770721009
	1050950	771149000
	1050738	770723007
	1047775	773692000
	1047616	776204008
	1046800	773587008
	1045479	773551001
	1044718	778011005
	1044090	782755007
	1041631	774102003
	1039010	783174004
	1038926	788417006
	1038153	783702009

	1036501	783005002
	1036205	774149004
	1036246	783175003
	1035656	770679002
	1035674	773274001
	1034910	773552008
	1033951	773405004
	1033797	778009001
	1033371	783089006
	1032195	782753000
	1032034	788584007
	1031732	771074000
	1031077	782723007
	1031103	782721009
	1031120	770750002
	1031042	787175002
	1030680	770898002
	1029366	773419004
	1028224	787174003
	1025789	773578004
	1025766	773772001
	1024316	783619003
	1022042	773553003
	1021562	773556006
	1021192	771148008
	1021122	773416006
	1020763	774068004
	1020209	770431001
	1018551	783703004
	1017416	773548008
	1017241	782945001
	1016970	782772000
	1013156	773581009
	1013191	774203000
	1012672	773583007
	1012490	773621003
	1012062	770901001
	1010211	773498006
	1009429	782736007
	1009470	771077007
	1008516	780827006
	1006179	770755007
	1065662	1173998003
	1077711	1260195002
	1059203	1172626003
	1061108	1187303004
	1061247	1169359006
	1061873	1186713004
	1062446	1220568003
	1062698	1217382002

	1062731	1172691004
	1063099	1186716007
	1063212	1186717003
	1063657	1208746001
	1064392	1186916004
	1065027	1186920000
	1065178	1220597000
	1065213	1217229007
	1065495	1186934000
	1066244	1172630000
	1066562	1208936008
	1067132	1172628002
	1067177	1186677001
	1067644	1187642008
	1068013	1197593006
	1068775	1208987006
	1068907	1186914001
	1069262	1197591008
	1069510	1187210007
	1069880	1169355000
	1070607	1186918003
	1070628	1186711002
	1070711	1179301003
	1070712	1177167002
	1072505	1187304005
	1073026	1172627007
	1073348	1187041000
	1073596	1217228004
	1073700	1187114007
	1074121	1172629005
	1074266	1186729007
	1074660	1167375003
	1074688	1172697000
	1074894	1186931008
	1075270	1208727002
	1075632	1179408008
	1075738	1187038009
	1076418	1222706005
	1076627	1172698005
	1078586	1237623009
	1078940	1255319004
	1078951	1260134001
	1079090	1237420004
	1079372	1260130005
	1080210	1254651003
	1080670	1255335006
	1080904	1254652005
	1081109	1254654006
	1082431	1254650002
	1091813	1269236003

	1097330	1269224009
	1097388	1284851009

TERM
CASK related intellectual disability
Syndromic X-linked intellectual disability due to JARID1C mutation
X-linked intellectual disability Cabezas type
X-linked intellectual disability Cantagrel type
FRAXE intellectual disability syndrome
Alpha thalassemia X-linked intellectual disability syndrome
X-linked intellectual disability with acromegaly and hyperactivity syndrome
X-linked intellectual disability Van Esch type
X-linked intellectual disability Stoll type
X-linked intellectual disability Turner type
Syndromic X-linked intellectual disability type 11
X-linked intellectual disability with ataxia and apraxia syndrome
Microcephalus, digital anomaly, intellectual disability syndrome
X-linked intellectual disability Shrimpton type
X-linked intellectual disability with corpus callosum agenesis and spastic quadriparesis syndrome
X-linked intellectual disability with cubitus valgus and dysmorphism syndrome
X-linked intellectual disability, limb spasticity, retinal dystrophy, diabetes insipidus syndrome
Laryngeal abductor paralysis with intellectual disability syndrome
Intellectual disability, cataract, calcified pinna, myopathy syndrome
X-linked intellectual disability Nascimento type
Hereditary congenital hypomelanotic and hypermelanotic cutaneous macules, growth retardation, intellectual
Non-progressive cerebellar ataxia with intellectual disability
X-linked intellectual disability, macrocephaly, macroorchidism syndrome
X-linked intellectual disability and hypotonia with facial dysmorphism and aggressive behaviour syndrome
Severe X-linked intellectual disability Gustavson type
Ectodermal dysplasia, intellectual disability, central nervous system malformation syndrome
Metaphyseal dysostosis, intellectual disability, conductive deafness syndrome
X-linked intellectual disability Stevenson type
X-linked intellectual disability Siderius type
X-linked intellectual disability Cilliers type
X-linked recessive intellectual disability and macrocephaly with ciliary dysfunction syndrome
Spastic tetraplegia, retinitis pigmentosa, intellectual disability syndrome
Syndromic X-linked intellectual disability type 7
X-linked intellectual disability Abidi type
X-linked intellectual disability Wilson type
Thumb stiffness, brachydactyly, intellectual disability syndrome
X-linked intellectual disability Seemanova type
Branchial dysplasia, intellectual disability, inguinal hernia syndrome
Fallot complex with intellectual disability and growth delay syndrome
Cortical blindness, intellectual disability, polydactyly syndrome
Intellectual disability Buenos Aires type
Hypotrichosis and intellectual disability syndrome Lopes type
X-linked intellectual disability Schimke type
Osteopenia, intellectual disability, sparse hair syndrome
X-linked intellectual disability with seizure and psoriasis syndrome
X-linked intellectual disability Miles Carpenter type
X-linked intellectual disability Brooks type
Alport syndrome, intellectual disability, midface hypoplasia, elliptocytosis syndrome
Spastic paraplegia, glaucoma, intellectual disability syndrome

X-linked intellectual disability and epilepsy with progressive joint contracture and facial dysmorphism syndrom
Megalocornea with intellectual disability syndrome
X-linked intellectual disability Zorick type
X-linked intellectual disability Armfield type
Preaxial polydactyly, colobomata, intellectual disability syndrome
Microcephaly, seizure, intellectual disability, heart disease syndrome
Ichthyosis, intellectual disability, dwarfism, renal impairment syndrome
X-linked intellectual disability with precocious puberty and obesity syndrome
X-linked intellectual disability Stocco Dos Santos type
X-linked intellectual disability with plagiocephaly syndrome
Severe intellectual disability, epilepsy, anal anomaly, distal phalangeal hypoplasia syndrome
Congenital hypoplasia of ulna and intellectual disability syndrome
X-linked intellectual disability with cerebellar hypoplasia syndrome
Marfanoid habitus with autosomal recessive intellectual disability syndrome
X-linked intellectual disability with hypogammaglobulinaemia and progressive neurological deterioration synd
Retinitis pigmentosa, intellectual disability, deafness, hypogenitalism syndrome
Coloboma, congenital heart disease, ichthyosiform dermatosis, intellectual disability ear anomaly syndrome
Seizures and intellectual disability due to hydroxylysinuria
X-linked spasticity, intellectual disability, epilepsy syndrome
X-linked intellectual disability Hedera type
Dentinogenesis imperfecta, short stature, hearing loss, intellectual disability syndrome
X-linked intellectual disability Pai type
Disorder of sex development with intellectual disability syndrome
Deafness and intellectual disability Martin Probst type syndrome
Intellectual disability with cataract and kyphosis syndrome
Pterygium colli with intellectual disability and digital anomaly syndrome
Alopecia and intellectual disability with hypergonadotropic hypogonadism syndrome
Hair defect with photosensitivity and intellectual disability syndrome
Osteogenesis imperfecta, retinopathy, seizures, intellectual disability syndrome
Early onset parkinsonism and intellectual disability syndrome
Aniridia and intellectual disability syndrome
Arachnodactyly and intellectual disability with facial dysmorphism syndrome
Skeletal dysplasia with intellectual disability syndrome
Agenesis of corpus callosum, intellectual disability, coloboma, micrognathia syndrome
Spondyloepiphyseal dysplasia, craniosynostosis, cleft palate, cataract and intellectual disability syndrome
Intellectual disability, developmental delay, contracture syndrome
Intellectual disability, epilepsy, bulbous nose syndrome
Intellectual disability, craniofacial dysmorphism, hypogonadism, diabetes mellitus syndrome
Congenital cataract with hypertrichosis and intellectual disability syndrome
Prune belly syndrome with pulmonic stenosis, intellectual disability and deafness
Osteopenia, myopia, hearing loss, intellectual disability, facial dysmorphism syndrome
Arachnodactyly with abnormal ossification and intellectual disability syndrome
Ataxia with deafness and intellectual disability syndrome
Uveal coloboma with cleft lip and palate and intellectual disability syndrome
Lipodystrophy, intellectual disability, deafness syndrome
Hypogonadism with mitral valve prolapse and intellectual disability syndrome
Alopecia, contracture, dwarfism, intellectual disability syndrome
Spastic paraplegia, intellectual disability, palmoplantar hyperkeratosis syndrome
Intellectual disability, hypoplastic corpus callosum, preauricular tag syndrome
Alopecia, psychomotor epilepsy, periodontal pyorrhoea, intellectual disability syndrome

Female restricted epilepsy with intellectual disability syndrome
Microphthalmia with ankyloblepharon and intellectual disability syndrome
Male hypergonadotropic hypogonadism, intellectual disability, skeletal anomaly syndrome
Microcephalus and intellectual disability with phalangeal and neurological anomaly syndrome
Seizure, sensorineural deafness, ataxia, intellectual disability, electrolyte imbalance syndrome
Aniridia, ptosis, intellectual disability, familial obesity syndrome
Family history of intellectual disability
Intellectual disability, myopathy, short stature, endocrine defect syndrome
Pachygyria, intellectual disability, epilepsy syndrome
Intellectual disability, brachydactyly, Pierre Robin syndrome
Intellectual disability Birk-Barel type
Intellectual disability, alacrima, achalasia syndrome
Intellectual disability, spasticity, ectrodactyly syndrome
Intellectual disability, congenital heart disease, blepharophimosis, blepharoptosis and hypoplastic teeth
Ichthyosis, alopecia, eclabion, ectropion, intellectual disability syndrome
X-linked intellectual disability with marfanoid habitus
ADNP-related multiple congenital anomalies, intellectual disability, autism spectrum disorder
X-linked intellectual disability, hypogonadism, ichthyosis, obesity, short stature syndrome
Alpha-thalassaemia intellectual disability syndrome linked to chromosome 16
Intellectual disability Wolff type
HIVEP2-related intellectual disability
Brachydactyly, mesomelia, intellectual disability, heart defect syndrome
Cerebellar ataxia, intellectual disability, oculomotor apraxia, cerebellar cysts syndrome
Craniodigital syndrome and intellectual disability syndrome
Intellectual disability screening
Aortic arch anomaly, facial dysmorphism, intellectual disability syndrome
Cryptorchidism, arachnodactyly, intellectual disability syndrome
Charcot-Marie-Tooth disease, deafness, intellectual disability syndrome
Hyperphosphatasaemia with intellectual disability
Intellectual disability due to nutritional deficiency
Germline WGS (whole genome sequencing) targeting intellectual disability panel
Focal epilepsy, intellectual disability, cerebro-cerebellar malformation syndrome
Intellectual disability, polydactyly, uncombable hair syndrome
Intellectual disability, obesity, brain malformation, facial dysmorphism syndrome
PPP2R5D-related intellectual disability
Intellectual disability germline WGS (whole genome sequencing) using microarray, fragile X and sequencing ta
X-linked intellectual disability hypotonic face syndrome
Microcephaly, thin corpus callosum, intellectual disability syndrome
Hepatic fibrosis, renal cyst, intellectual disability syndrome
Optic atrophy, intellectual disability syndrome
Late-onset localized junctional epidermolysis bullosa, intellectual disability syndrome
Colobomatous microphthalmia, obesity, hypogenitalism, intellectual disability syndrome
X-linked intellectual disability, cardiomegaly, congestive heart failure syndrome
Severe intellectual disability, poor language, strabismus, grimacing face, long fingers syndrome
Severe intellectual disability and progressive spastic paraplegia
Primary microcephaly, mild intellectual disability, young-onset diabetes syndrome
Intellectual disability, obesity, prognathism, eye and skin anomalies syndrome
Congenital muscular dystrophy with intellectual disability
Alopecia, epilepsy, intellectual disability syndrome Moynahan type
X-linked intellectual disability due to GRIA3 mutations

Severe microbrachycephaly, intellectual disability, athetoid cerebral palsy syndrome
Severe intellectual disability, progressive postnatal microcephaly, midline stereotypic hand movements syndro
Congenital muscular dystrophy without intellectual disability
Polyneuropathy, intellectual disability, acromicria, premature menopause syndrome
X-linked intellectual disability, craniofacioskeletal syndrome
Intellectual disability, feeding difficulties, developmental delay, microcephaly syndrome
Intellectual disability with strabismus syndrome
Blepharophimosis, intellectual disability syndrome, Verloes type
Macrocephaly, intellectual disability, autism syndrome
Intellectual disability, coarse face, macrocephaly, cerebellar hypotrophy syndrome
Blepharophimosis, intellectual disability syndrome
Microcephaly, short stature, intellectual disability, facial dysmorphism syndrome
Severe intellectual disability, progressive spastic diplegia syndrome
Autosomal recessive cerebellar ataxia, epilepsy, intellectual disability syndrome due to RUBCN deficiency
Intellectual disability, seizures, macrocephaly, obesity syndrome
ANK3-related intellectual disability, sleep disturbance syndrome
Autosomal recessive cerebellar ataxia, epilepsy, intellectual disability syndrome due to WWOX deficiency
Severe intellectual disability, short stature, behavioural abnormalities, facial dysmorphism syndrome
Intellectual disability, hyperkinetic movement, truncal ataxia syndrome
Spondylocostal dysostosis, hypospadias, intellectual disability syndrome
Rare non-syndromic intellectual disability
DYRK1A-related intellectual disability syndrome due to 21q22.13q22.2 microdeletion
Hypohidrosis, enamel hypoplasia, palmoplantar keratoderma, intellectual disability syndrome
Short ulna, dysmorphism, hypotonia, intellectual disability syndrome
X-linked colobomatous microphthalmia, microcephaly, intellectual disability, short stature syndrome
Intellectual disability, facial dysmorphism, hand anomalies syndrome
AHDC1-related intellectual disability, obstructive sleep apnoea, mild dysmorphism syndrome
Early-onset epileptic encephalopathy and intellectual disability due to GRIN2A mutation
White matter hypoplasia, corpus callosum agenesis, intellectual disability syndrome
Early-onset epileptic encephalopathy, cortical blindness, intellectual disability, facial dysmorphism syndrome
Ophthalmoplegia, intellectual disability, lingua scrotalis syndrome
Congenital muscular dystrophy with intellectual disability and severe epilepsy
Intellectual disability, craniofacial dysmorphism, cryptorchidism syndrome
Intellectual disability, severe speech delay, mild dysmorphism syndrome
Aphonia, deafness, retinal dystrophy, bifid halluces, intellectual disability syndrome
Intellectual disability, hypotonia, brachycephaly, pyloric stenosis, cryptorchidism syndrome
Autosomal recessive intellectual disability, motor dysfunction, multiple joint contracture syndrome
Autosomal recessive cerebellar ataxia, epilepsy, intellectual disability syndrome due to TUD deficiency
Intellectual disability, facial dysmorphism syndrome due to SETD5 haploinsufficiency
Intellectual disability, short stature, hypertelorism syndrome
SYNGAP1-related intellectual disability
Intellectual disability, seizures, hypotonia, ophthalmologic, skeletal anomalies syndrome
Postnatal microcephaly, infantile hypotonia, spastic diplegia, dysarthria, intellectual disability syndrome
GRIN2B-related developmental delay, intellectual disability, autism spectrum disorder
TELO2-related intellectual disability, neurodevelopmental disorder
Progressive spondyloepimetaphyseal dysplasia, short stature, short fourth metatarsals, intellectual disability s
Tall stature, intellectual disability, renal anomalies syndrome
Growth delay, intellectual disability, hepatopathy syndrome
QRICH1-related intellectual disability, chondrodysplasia syndrome
Intellectual disability, autism, speech apraxia, craniofacial dysmorphism syndrome

CLCN4-related X-linked intellectual disability syndrome
Intellectual disability psychiatrist
Intellectual disability psychiatry service
Intellectual disability, muscle weakness, short stature, facial dysmorphism syndrome
Intellectual disability psychiatry department
Referral to intellectual disability psychiatry service
Retinitis pigmentosa, juvenile cataract, short stature, intellectual disability syndrome
Craniofacial dysplasia, short stature, ectodermal anomalies, intellectual disability syndrome
Caregiver of dependent with intellectual disability
Global developmental delay, neuro-ophthalmological abnormalities, seizures, intellectual disability syndrome
Congenital ichthyosis, intellectual disability, spastic quadriplegia syndrome
TBCK-related intellectual disability syndrome
Intellectual disability specialty
Macrocephaly, intellectual disability, left ventricular non compaction syndrome
Intellectual disability, expressive aphasia, facial dysmorphism syndrome
PHIP-related behavioural problems, intellectual disability, obesity, dysmorphic features syndrome
Intellectual disability nurse
Severe intellectual disability, hypotonia, strabismus, coarse face, planovalgus syndrome
Intellectual disability, epilepsy, extrapyramidal syndrome
Brain malformations, musculoskeletal abnormalities, facial dysmorphism, intellectual disability syndrome
Referral to intellectual disability psychiatrist
GNB5-related intellectual disability, cardiac arrhythmia syndrome
DYRK1A-related intellectual disability syndrome
Intellectual disability, seizures, abnormal gait, facial dysmorphism syndrome
Macrocephaly, intellectual disability, neurodevelopmental disorder, small thorax syndrome
Early-onset epilepsy, intellectual disability, brain anomalies syndrome
STAG1-related intellectual disability, facial dysmorphism, gastrooesophageal reflux syndrome
X-linked intellectual disability, cerebellar hypoplasia, spondyloepiphyseal dysplasia syndrome
Micrognathia, recurrent infections, behavioural abnormalities, mild intellectual disability syndrome
Severe growth deficiency, strabismus, extensive dermal melanocytosis, intellectual disability syndrome
Intellectual disability, cardiac anomalies, short stature, joint laxity syndrome
Microcephaly, corpus callosum and cerebellar vermis hypoplasia, facial dysmorphism, intellectual disability syı
X-linked female restricted facial dysmorphism, short stature, choanal atresia, intellectual disability
Intellectual disability teacher
Severe intellectual disability, agenesis of corpus callosum, facial dysmorphism, cerebellar ataxia syndrome
CHD3-related developmental delay, speech delay, intellectual disability, abnormalities of vision, facial dysmor
Non-specific syndromic intellectual disability
Anterior maxillary protrusion, strabismus, intellectual disability syndrome
Recurrent metabolic encephalomyopathic crises, rhabdomyolysis, cardiac arrhythmia, intellectual disability syı
Congenital insensitivity to pain with severe intellectual disability
Autosomal dominant intellectual disability, craniofacial anomalies, cardiac defects syndrome
Spastic paraplegia, intellectual disability, nystagmus, obesity syndrome
X-linked intellectual disability, global development delay, facial dysmorphism, sacral caudal remnant syndrome
Progressive essential tremor, speech impairment, facial dysmorphism, intellectual disability, abnormal behavic
Microcephaly, intellectual disability, sensorineural hearing loss, epilepsy, abnormal muscle tone syndrome
X-linked intellectual disability, short stature, overweight syndrome
Intellectual disability, macrocephaly, hypotonia, behavioural abnormalities syndrome
X-linked intellectual disability, hypotonia, movement disorder syndrome
Microcephaly, corpus callosum hypoplasia, intellectual disability, facial dysmorphism syndrome
Primary hypomagnesemia, refractory seizures, intellectual disability syndrome

Craniosynostosis, microretrognathia, severe intellectual disability syndrome
Short stature, skeletal dysplasia, retinal degeneration, intellectual disability, sensorineural hearing loss syndro[illegible]

rgeting intellectual disability panel
