# Supplementary material for: The overlooked burden: anti-seizure medications, laxatives, and antipsychotics prescribed in primary care for people with intellectual disability
Source: Front Psychiatry. 2026 Feb 19;17:1714524. doi: 10.3389/fpsyt.2026.1714524 (PMC12960475; doi:10.3389/fpsyt.2026.1714524)
Supplement: Supplementary file 5 [file DataSheet2.pdf]

SKEY_GPD_QOF_GROUPS	SKEY_SNOMED_CONCEPT	CLUSTER_ID
23249	653	LD_COD
23250	3196	LD_COD
23251	9105	LD_COD
23254	33884	LD_COD
23260	271587	LD_COD
23088	390243	LD_COD
23268	415815	LD_COD
23267	420847	LD_COD
23090	436981	LD_COD
23411	450447	LD_COD
23229	458449	LD_COD
23255	481930	LD_COD
23412	486864	LD_COD
23124	487450	LD_COD
23285	488067	LD_COD
23096	489656	LD_COD
23100	489850	LD_COD
23270	491236	LD_COD
23269	491379	LD_COD
23292	493071	LD_COD
23095	493894	LD_COD
23097	494742	LD_COD
23274	496006	LD_COD
23317	498331	LD_COD
23277	498785	LD_COD
23098	499591	LD_COD
23281	500269	LD_COD
23131	500306	LD_COD
23272	501107	LD_COD
23278	501619	LD_COD
23276	501966	LD_COD
23275	505077	LD_COD
23290	505364	LD_COD
23108	505987	LD_COD
23289	507703	LD_COD
23105	507864	LD_COD
23306	510349	LD_COD
23171	511891	LD_COD
23106	512012	LD_COD
23119	512741	LD_COD
23301	513114	LD_COD
23296	513423	LD_COD
23315	514777	LD_COD
23342	515093	LD_COD
23313	515116	LD_COD
23297	515121	LD_COD
23347	515273	LD_COD
23123	515644	LD_COD
23165	516369	LD_COD

23159	516819	LD_COD
23166	516835	LD_COD
23337	517127	LD_COD
23117	517190	LD_COD
23145	517630	LD_COD
23141	517921	LD_COD
23318	518194	LD_COD
23177	518500	LD_COD
23355	518533	LD_COD
23287	518595	LD_COD
23107	519107	LD_COD
23291	519109	LD_COD
23333	519125	LD_COD
23164	519524	LD_COD
23114	520498	LD_COD
23115	520596	LD_COD
23338	520753	LD_COD
23153	520880	LD_COD
23101	521246	LD_COD
23280	521373	LD_COD
23300	521667	LD_COD
23294	521691	LD_COD
23109	522572	LD_COD
23304	522788	LD_COD
23354	522806	LD_COD
23286	522967	LD_COD
23350	523124	LD_COD
23151	523572	LD_COD
23348	523896	LD_COD
23351	524404	LD_COD
23161	524831	LD_COD
23152	525480	LD_COD
23325	525592	LD_COD
23324	526023	LD_COD
23126	526240	LD_COD
23103	526286	LD_COD
23110	528334	LD_COD
23349	528464	LD_COD
23305	528752	LD_COD
23112	528817	LD_COD
23163	528887	LD_COD
23143	529059	LD_COD
23326	529336	LD_COD
23321	529339	LD_COD
23316	529366	LD_COD
23174	529883	LD_COD
23343	529903	LD_COD
23173	529924	LD_COD
23129	530093	LD_COD
23271	530102	LD_COD

23116	530105 LD_COD
23168	530235 LD_COD
23176	531318 LD_COD
23314	531788 LD_COD
23154	531800 LD_COD
23284	531833 LD_COD
23273	531986 LD_COD
23293	532707 LD_COD
23170	533100 LD_COD
23149	533131 LD_COD
23172	533317 LD_COD
23322	533593 LD_COD
23113	534185 LD_COD
23307	534246 LD_COD
23288	534492 LD_COD
23125	534493 LD_COD
23299	534529 LD_COD
23150	535118 LD_COD
23156	535263 LD_COD
23158	535492 LD_COD
23155	536047 LD_COD
23167	536162 LD_COD
23169	536380 LD_COD
23341	536434 LD_COD
23353	537244 LD_COD
23139	538782 LD_COD
23127	541286 LD_COD
23295	542300 LD_COD
23266	544013 LD_COD
23336	544111 LD_COD
23157	545172 LD_COD
23130	547062 LD_COD
23339	547315 LD_COD
23160	547706 LD_COD
23352	547769 LD_COD
23346	548535 LD_COD
23137	549281 LD_COD
23175	549321 LD_COD
23111	549767 LD_COD
23279	550272 LD_COD
23340	551858 LD_COD
23298	553596 LD_COD
23122	554613 LD_COD
23410	555071 LD_COD
23344	555088 LD_COD
23162	555509 LD_COD
23136	555523 LD_COD
23140	555984 LD_COD
23345	565971 LD_COD
23133	569050 LD_COD

23134	569412	LD_COD
23257	574755	LD_COD
23144	578578	LD_COD
23120	585264	LD_COD
23128	594527	LD_COD
23138	607023	LD_COD
23086	612595	LD_COD
23244	612853	LD_COD
23118	613570	LD_COD
23148	626245	LD_COD
23283	626641	LD_COD
23331	627037	LD_COD
23093	647978	LD_COD
23099	649615	LD_COD
23102	652190	LD_COD
23104	653246	LD_COD
23335	653447	LD_COD
23261	655533	LD_COD
23081	668129	LD_COD
23319	670664	LD_COD
23334	673385	LD_COD
23282	673445	LD_COD
23332	682805	LD_COD
23328	684185	LD_COD
23309	688442	LD_COD
23094	689392	LD_COD
23310	692367	LD_COD
23323	696750	LD_COD
23308	701745	LD_COD
23302	711558	LD_COD
23092	718060	LD_COD
23089	718903	LD_COD
23132	725473	LD_COD
23329	727004	LD_COD
23247	727319	LD_COD
23246	731964	LD_COD
23074	738144	LD_COD
23146	742223	LD_COD
23075	747473	LD_COD
23327	773412	LD_COD
23135	773993	LD_COD
23259	776783	LD_COD
23147	791864	LD_COD
23080	795608	LD_COD
23091	799569	LD_COD
23330	809736	LD_COD
23311	809913	LD_COD
23121	813317	LD_COD
23312	814365	LD_COD
23320	815590	LD_COD

23264	823041	LD_COD
23142	827354	LD_COD
23303	827433	LD_COD
23065	835168	LD_COD
23060	840273	LD_COD
23186	841798	LD_COD
23189	842892	LD_COD
23068	844600	LD_COD
23059	844679	LD_COD
23237	844997	LD_COD
23366	845015	LD_COD
23064	846031	LD_COD
23066	846352	LD_COD
23370	846563	LD_COD
23187	847264	LD_COD
23362	848099	LD_COD
23188	850995	LD_COD
23185	853718	LD_COD
23239	853778	LD_COD
23361	853938	LD_COD
23069	854000	LD_COD
23070	855132	LD_COD
23063	855156	LD_COD
23078	855776	LD_COD
23258	855826	LD_COD
23181	856243	LD_COD
23357	856663	LD_COD
23082	857197	LD_COD
23193	857937	LD_COD
23178	857972	LD_COD
23363	858542	LD_COD
23192	858545	LD_COD
23194	859007	LD_COD
23084	860042	LD_COD
23356	860057	LD_COD
23405	861992	LD_COD
23236	862577	LD_COD
23061	862650	LD_COD
23191	863515	LD_COD
23062	863817	LD_COD
23079	865416	LD_COD
23067	865724	LD_COD
23184	866528	LD_COD
23182	868290	LD_COD
23367	869625	LD_COD
23179	869649	LD_COD
23253	870780	LD_COD
23240	871088	LD_COD
23183	871372	LD_COD
23265	872910	LD_COD

23190	873569 LD_COD
23359	874800 LD_COD
23241	875388 LD_COD
23360	877228 LD_COD
23369	877516 LD_COD
23180	878229 LD_COD
23238	878793 LD_COD
23364	878956 LD_COD
23195	879019 LD_COD
23057	879131 LD_COD
23365	879573 LD_COD
23243	880468 LD_COD
23242	881496 LD_COD
23358	909491 LD_COD
23087	935621 LD_COD
23256	936092 LD_COD
23262	939702 LD_COD
23077	951042 LD_COD
23085	953502 LD_COD
23071	954157 LD_COD
23263	956290 LD_COD
23368	958663 LD_COD
23248	959555 LD_COD
23073	962910 LD_COD
23083	963065 LD_COD
23403	969665 LD_COD
23228	975545 LD_COD
23196	978626 LD_COD
23072	980939 LD_COD
23252	990491 LD_COD
23076	995164 LD_COD
23230	1006609 LD_COD
23377	1050950 LD_COD
23245	1050280 LD_COD
23234	1049521 LD_COD
23383	1048503 LD_COD
23208	1047263 LD_COD
23371	1047400 LD_COD
23406	1046714 LD_COD
23227	1046623 LD_COD
23404	1046112 LD_COD
23394	1044718 LD_COD
23398	1044090 LD_COD
23376	1043903 LD_COD
23375	1042884 LD_COD
23391	1040500 LD_COD
23199	1039256 LD_COD
23224	1039327 LD_COD
23395	1039125 LD_COD
23402	1039010 LD_COD

23216	1038926	LD_COD
23231	1039081	LD_COD
23213	1038153	LD_COD
23389	1037611	LD_COD
23384	1037048	LD_COD
23401	1036501	LD_COD
23201	1035656	LD_COD
23205	1035523	LD_COD
23378	1034479	LD_COD
23385	1033951	LD_COD
23400	1033818	LD_COD
23390	1032801	LD_COD
23397	1032195	LD_COD
23379	1031483	LD_COD
23210	1031077	LD_COD
23202	1031120	LD_COD
23221	1029965	LD_COD
23209	1029619	LD_COD
23218	1029474	LD_COD
23386	1029366	LD_COD
23058	1028979	LD_COD
23222	1028784	LD_COD
23215	1026113	LD_COD
23200	1025980	LD_COD
23392	1025766	LD_COD
23374	1024096	LD_COD
23373	1023636	LD_COD
23235	1023773	LD_COD
23382	1023170	LD_COD
23393	1020763	LD_COD
23409	1019888	LD_COD
23223	1019422	LD_COD
23214	1018551	LD_COD
23207	1018439	LD_COD
23207	1018439	LD_COD
23212	1017241	LD_COD
23211	1017050	LD_COD
23399	1016970	LD_COD
23380	1016631	LD_COD
23220	1016149	LD_COD
23217	1015883	LD_COD
23206	1015578	LD_COD
23388	1013156	LD_COD
23233	1012680	LD_COD
23204	1012062	LD_COD
23407	1011780	LD_COD
23226	1011584	LD_COD
23387	1010211	LD_COD
23197	1009901	LD_COD
23396	1009429	LD_COD

23219	1007744	LD_COD
23203	1006179	LD_COD
23372	1005200	LD_COD
23232	1004938	LD_COD
23198	1004708	LD_COD
23225	1004836	LD_COD
23381	1004757	LD_COD
23408	1004543	LD_COD

CLUSTER_DESCRIPTION	SNOMED_CONCEPT_ID
Learning disability (LD) codes	232059000
Learning disability (LD) codes	234146006
Learning disability (LD) codes	253176002
Learning disability (LD) codes	371045000
Learning disability (LD) codes	5.08171E+14
Learning disability (LD) codes	699298009
Learning disability (LD) codes	702412005
Learning disability (LD) codes	702344008
Learning disability (LD) codes	702327009
Learning disability (LD) codes	9.84671E+14
Learning disability (LD) codes	9.31001E+14
Learning disability (LD) codes	401315004
Learning disability (LD) codes	9.84681E+14
Learning disability (LD) codes	719811001
Learning disability (LD) codes	718848000
Learning disability (LD) codes	715989002
Learning disability (LD) codes	717822006
Learning disability (LD) codes	703535000
Learning disability (LD) codes	702416008
Learning disability (LD) codes	719016007
Learning disability (LD) codes	715628009
Learning disability (LD) codes	716089008
Learning disability (LD) codes	716112005
Learning disability (LD) codes	721008000
Learning disability (LD) codes	716996008
Learning disability (LD) codes	716334004
Learning disability (LD) codes	718226002
Learning disability (LD) codes	720957007
Learning disability (LD) codes	716024001
Learning disability (LD) codes	717223008
Learning disability (LD) codes	716709002
Learning disability (LD) codes	716191002
Learning disability (LD) codes	718914002
Learning disability (LD) codes	718911005
Learning disability (LD) codes	718912003
Learning disability (LD) codes	718900002
Learning disability (LD) codes	719909009
Learning disability (LD) codes	733090001
Learning disability (LD) codes	718905007
Learning disability (LD) codes	719202006
Learning disability (LD) codes	719212004
Learning disability (LD) codes	719137001
Learning disability (LD) codes	720955004
Learning disability (LD) codes	725289009
Learning disability (LD) codes	720748007
Learning disability (LD) codes	719138006
Learning disability (LD) codes	732246009
Learning disability (LD) codes	719808002
Learning disability (LD) codes	726709001

Learning disability (LD) codes	725140007
Learning disability (LD) codes	726732002
Learning disability (LD) codes	723441001
Learning disability (LD) codes	719157002
Learning disability (LD) codes	722213009
Learning disability (LD) codes	722031003
Learning disability (LD) codes	721017000
Learning disability (LD) codes	734017008
Learning disability (LD) codes	733419006
Learning disability (LD) codes	718909001
Learning disability (LD) codes	718908009
Learning disability (LD) codes	719013004
Learning disability (LD) codes	722380003
Learning disability (LD) codes	726031001
Learning disability (LD) codes	719069008
Learning disability (LD) codes	719097002
Learning disability (LD) codes	723621000
Learning disability (LD) codes	723403008
Learning disability (LD) codes	717887003
Learning disability (LD) codes	717945001
Learning disability (LD) codes	719160009
Learning disability (LD) codes	719018008
Learning disability (LD) codes	719009006
Learning disability (LD) codes	719800009
Learning disability (LD) codes	733117001
Learning disability (LD) codes	718897009
Learning disability (LD) codes	732961003
Learning disability (LD) codes	723336008
Learning disability (LD) codes	732251003
Learning disability (LD) codes	733031004
Learning disability (LD) codes	725906006
Learning disability (LD) codes	723365002
Learning disability (LD) codes	722035007
Learning disability (LD) codes	721974000
Learning disability (LD) codes	719834005
Learning disability (LD) codes	718577005
Learning disability (LD) codes	719010001
Learning disability (LD) codes	732954002
Learning disability (LD) codes	719810000
Learning disability (LD) codes	719012009
Learning disability (LD) codes	725912001
Learning disability (LD) codes	722065002
Learning disability (LD) codes	722037004
Learning disability (LD) codes	721224008
Learning disability (LD) codes	720982007
Learning disability (LD) codes	733455003
Learning disability (LD) codes	725589005
Learning disability (LD) codes	733417008
Learning disability (LD) codes	720523006
Learning disability (LD) codes	715409005

Learning disability (LD) codes	719155005
Learning disability (LD) codes	733049004
Learning disability (LD) codes	733522005
Learning disability (LD) codes	720954000
Learning disability (LD) codes	723501008
Learning disability (LD) codes	718846001
Learning disability (LD) codes	716096005
Learning disability (LD) codes	719017003
Learning disability (LD) codes	733088002
Learning disability (LD) codes	723304001
Learning disability (LD) codes	733097003
Learning disability (LD) codes	721875000
Learning disability (LD) codes	719020006
Learning disability (LD) codes	719947004
Learning disability (LD) codes	718910006
Learning disability (LD) codes	719812008
Learning disability (LD) codes	719140001
Learning disability (LD) codes	723332005
Learning disability (LD) codes	723676007
Learning disability (LD) codes	724137002
Learning disability (LD) codes	723504000
Learning disability (LD) codes	733032006
Learning disability (LD) codes	733086003
Learning disability (LD) codes	724228005
Learning disability (LD) codes	733110004
Learning disability (LD) codes	722002002
Learning disability (LD) codes	719842006
Learning disability (LD) codes	719136005
Learning disability (LD) codes	70173007
Learning disability (LD) codes	723333000
Learning disability (LD) codes	724001005
Learning disability (LD) codes	720639008
Learning disability (LD) codes	723994004
Learning disability (LD) codes	725163002
Learning disability (LD) codes	733072002
Learning disability (LD) codes	726727003
Learning disability (LD) codes	721089006
Learning disability (LD) codes	733472005
Learning disability (LD) codes	719011002
Learning disability (LD) codes	717763008
Learning disability (LD) codes	724207001
Learning disability (LD) codes	719139003
Learning disability (LD) codes	719450007
Learning disability (LD) codes	9.84661E+14
Learning disability (LD) codes	726670008
Learning disability (LD) codes	725908007
Learning disability (LD) codes	721087008
Learning disability (LD) codes	722003007
Learning disability (LD) codes	726672000
Learning disability (LD) codes	720981000

Learning disability (LD) codes	721007005
Learning disability (LD) codes	403554008
Learning disability (LD) codes	722110003
Learning disability (LD) codes	719378009
Learning disability (LD) codes	720468000
Learning disability (LD) codes	721208007
Learning disability (LD) codes	66758006
Learning disability (LD) codes	110359009
Learning disability (LD) codes	719162001
Learning disability (LD) codes	722478008
Learning disability (LD) codes	718681002
Learning disability (LD) codes	722282008
Learning disability (LD) codes	703526007
Learning disability (LD) codes	717157006
Learning disability (LD) codes	718573009
Learning disability (LD) codes	718766002
Learning disability (LD) codes	722456001
Learning disability (LD) codes	51500006
Learning disability (LD) codes	416075005
Learning disability (LD) codes	721146009
Learning disability (LD) codes	722454003
Learning disability (LD) codes	718680001
Learning disability (LD) codes	722379001
Learning disability (LD) codes	722111004
Learning disability (LD) codes	720501007
Learning disability (LD) codes	715428003
Learning disability (LD) codes	720517001
Learning disability (LD) codes	721973006
Learning disability (LD) codes	720401009
Learning disability (LD) codes	719380003
Learning disability (LD) codes	702816000
Learning disability (LD) codes	699669001
Learning disability (LD) codes	720979002
Learning disability (LD) codes	722209002
Learning disability (LD) codes	205616004
Learning disability (LD) codes	17122004
Learning disability (LD) codes	205615000
Learning disability (LD) codes	722455002
Learning disability (LD) codes	21111006
Learning disability (LD) codes	722055008
Learning disability (LD) codes	721073008
Learning disability (LD) codes	442511009
Learning disability (LD) codes	722459008
Learning disability (LD) codes	41040004
Learning disability (LD) codes	702357000
Learning disability (LD) codes	722281001
Learning disability (LD) codes	720635002
Learning disability (LD) codes	719396000
Learning disability (LD) codes	720746006
Learning disability (LD) codes	721207002

Learning disability (LD) codes	59252009
Learning disability (LD) codes	722033000
Learning disability (LD) codes	719599008
Learning disability (LD) codes	1.08978E+15
Learning disability (LD) codes	1.08972E+15
Learning disability (LD) codes	763773007
Learning disability (LD) codes	764959000
Learning disability (LD) codes	1.09399E+15
Learning disability (LD) codes	1.0897E+15
Learning disability (LD) codes	1.08977E+15
Learning disability (LD) codes	763861000
Learning disability (LD) codes	1.08976E+15
Learning disability (LD) codes	1.08979E+15
Learning disability (LD) codes	766871009
Learning disability (LD) codes	763795006
Learning disability (LD) codes	763744009
Learning disability (LD) codes	764861005
Learning disability (LD) codes	763741001
Learning disability (LD) codes	1.08982E+15
Learning disability (LD) codes	763743003
Learning disability (LD) codes	1.094E+15
Learning disability (LD) codes	1.09401E+15
Learning disability (LD) codes	1.08975E+15
Learning disability (LD) codes	31216003
Learning disability (LD) codes	412787009
Learning disability (LD) codes	763404001
Learning disability (LD) codes	763320005
Learning disability (LD) codes	422437002
Learning disability (LD) codes	765471005
Learning disability (LD) codes	734349003
Learning disability (LD) codes	763745005
Learning disability (LD) codes	765434008
Learning disability (LD) codes	765761009
Learning disability (LD) codes	61152003
Learning disability (LD) codes	763186006
Learning disability (LD) codes	86765009
Learning disability (LD) codes	1.08971E+15
Learning disability (LD) codes	1.08973E+15
Learning disability (LD) codes	765170001
Learning disability (LD) codes	1.08974E+15
Learning disability (LD) codes	40700009
Learning disability (LD) codes	1.08985E+15
Learning disability (LD) codes	763665007
Learning disability (LD) codes	763615003
Learning disability (LD) codes	764950001
Learning disability (LD) codes	763136000
Learning disability (LD) codes	33982008
Learning disability (LD) codes	1.08983E+15
Learning disability (LD) codes	763626009
Learning disability (LD) codes	699297004

Learning disability (LD) codes	765089003
Learning disability (LD) codes	763722004
Learning disability (LD) codes	1.08984E+15
Learning disability (LD) codes	763742008
Learning disability (LD) codes	766870005
Learning disability (LD) codes	763350002
Learning disability (LD) codes	1.08981E+15
Learning disability (LD) codes	763797003
Learning disability (LD) codes	768677000
Learning disability (LD) codes	10007009
Learning disability (LD) codes	763837007
Learning disability (LD) codes	1.09403E+15
Learning disability (LD) codes	1.09402E+15
Learning disability (LD) codes	763618001
Learning disability (LD) codes	68618008
Learning disability (LD) codes	40354009
Learning disability (LD) codes	5619004
Learning disability (LD) codes	2593002
Learning disability (LD) codes	65327002
Learning disability (LD) codes	109478007
Learning disability (LD) codes	56604005
Learning disability (LD) codes	766753005
Learning disability (LD) codes	21634003
Learning disability (LD) codes	17827007
Learning disability (LD) codes	57917004
Learning disability (LD) codes	79385002
Learning disability (LD) codes	89392001
Learning disability (LD) codes	76880004
Learning disability (LD) codes	15182000
Learning disability (LD) codes	254268004
Learning disability (LD) codes	254264002
Learning disability (LD) codes	1003368009
Learning disability (LD) codes	771149000
Learning disability (LD) codes	1.23933E+15
Learning disability (LD) codes	1003409002
Learning disability (LD) codes	773394007
Learning disability (LD) codes	773230003
Learning disability (LD) codes	770401007
Learning disability (LD) codes	879937000
Learning disability (LD) codes	890286007
Learning disability (LD) codes	816067005
Learning disability (LD) codes	778011005
Learning disability (LD) codes	782755007
Learning disability (LD) codes	770948004
Learning disability (LD) codes	770908007
Learning disability (LD) codes	773769008
Learning disability (LD) codes	770595006
Learning disability (LD) codes	890130000
Learning disability (LD) codes	782676009
Learning disability (LD) codes	783174004

Learning disability (LD) codes	788417006
Learning disability (LD) codes	1003373003
Learning disability (LD) codes	783702009
Learning disability (LD) codes	773670004
Learning disability (LD) codes	773400009
Learning disability (LD) codes	783005002
Learning disability (LD) codes	770679002
Learning disability (LD) codes	770907002
Learning disability (LD) codes	771262009
Learning disability (LD) codes	773405004
Learning disability (LD) codes	782886007
Learning disability (LD) codes	773699009
Learning disability (LD) codes	782753000
Learning disability (LD) codes	771336003
Learning disability (LD) codes	782723007
Learning disability (LD) codes	770750002
Learning disability (LD) codes	880081006
Learning disability (LD) codes	773665006
Learning disability (LD) codes	840505007
Learning disability (LD) codes	773419004
Learning disability (LD) codes	1003387003
Learning disability (LD) codes	890118006
Learning disability (LD) codes	787093004
Learning disability (LD) codes	770663003
Learning disability (LD) codes	773772001
Learning disability (LD) codes	770719004
Learning disability (LD) codes	770678005
Learning disability (LD) codes	1010630006
Learning disability (LD) codes	773329005
Learning disability (LD) codes	774068004
Learning disability (LD) codes	890434000
Learning disability (LD) codes	890123006
Learning disability (LD) codes	783703004
Learning disability (LD) codes	771512003
Learning disability (LD) codes	771512003
Learning disability (LD) codes	782945001
Learning disability (LD) codes	782941005
Learning disability (LD) codes	782772000
Learning disability (LD) codes	771472009
Learning disability (LD) codes	880066000
Learning disability (LD) codes	838441009
Learning disability (LD) codes	771072001
Learning disability (LD) codes	773581009
Learning disability (LD) codes	1003389000
Learning disability (LD) codes	770901001
Learning disability (LD) codes	880065001
Learning disability (LD) codes	890285006
Learning disability (LD) codes	773498006
Learning disability (LD) codes	770564004
Learning disability (LD) codes	782736007

Learning disability (LD) codes	879919001
Learning disability (LD) codes	770755007
Learning disability (LD) codes	770411000
Learning disability (LD) codes	1003374009
Learning disability (LD) codes	770566002
Learning disability (LD) codes	890221004
Learning disability (LD) codes	772127009
Learning disability (LD) codes	890433006

TERM
Laurence-Moon syndrome
Hennekam syndrome
Gillespie syndrome
Translocation Down syndrome
Severe learning disability
Ohdo syndrome, Say-Barber-Biesecker-Young-Simpson variant
Partington syndrome
Pitt-Hopkins syndrome
Allan-Herndon-Dudley syndrome
Moderate learning disability
Significant learning disability
Smith-Magenis syndrome
Profound learning disability
X-linked intellectual disability Cabezas type
Fried syndrome
Karandikar Maria Kamble syndrome
Goldberg Shprintzen megacolon syndrome
Mowat-Wilson syndrome
Snyder-Robinson syndrome
X-linked intellectual disability Cantagrel type
MORM syndrome
Harrod syndrome
Kawashima Tsuji syndrome
Hall Riggs syndrome
L1 syndrome
Urban Rogers Meyer syndrome
Wolf Hirschhorn syndrome
Fountain syndrome
GMS syndrome
X-linked epilepsy with learning disability and behaviour disorder syndrome
FRAXE intellectual disability syndrome
Perniola Krajewska Carnevale syndrome
X-linked intellectual disability Van Esch type
X-linked intellectual disability Stoll type
X-linked intellectual disability Turner type
Syndromic X-linked intellectual disability type 11
Trisomy Xq28
Microcephalus, digital anomaly, intellectual disability syndrome
X-linked intellectual disability Shrimpton type
Spondyloepiphyseal dysplasia tarda Kohn type
Smith Fineman Myers syndrome
X-linked intellectual disability with corpus callosum agenesis and spastic quadriparesis syndrome
Fine Lubinsky syndrome
5-amino-4-imidazole carboxamide ribosiduria
Cooper Jabs syndrome
X-linked intellectual disability with cubitus valgus and dysmorphism syndrome
X-linked intellectual disability, limb spasticity, retinal dystrophy, diabetes insipidus syndrome
Chromosome Xp11.3 microdeletion syndrome
Intellectual disability, cataract, calcified pinna, myopathy syndrome

Temple Baraitser syndrome
X-linked intellectual disability Nascimento type
Non-progressive cerebellar ataxia with intellectual disability
X-linked intellectual disability and hypotonia with facial dysmorphism and aggressive behaviour syndrome
Severe X-linked intellectual disability Gustavson type
Kapur Toriello syndrome
Oliver syndrome
Ectodermal dysplasia, intellectual disability, central nervous system malformation syndrome
Metaphyseal dysostosis, intellectual disability, conductive deafness syndrome
X-linked intellectual disability Stevenson type
X-linked intellectual disability Siderius type
X-linked intellectual disability Cilliers type
Martsolf syndrome
CAMOS syndrome
Shprintzen Goldberg craniosynostosis syndrome
BSG syndrome
Spastic tetraplegia, retinitis pigmentosa, intellectual disability syndrome
Microbrachycephaly, ptosis, cleft lip syndrome
Biemond syndrome type 2
BRESEK syndrome
Syndromic X-linked intellectual disability type 7
X-linked intellectual disability Abidi type
X-linked intellectual disability Wilson type
DOORS syndrome
Thumb stiffness, brachydactyly, intellectual disability syndrome
X-linked intellectual disability Seemanova type
Branchial dysplasia, intellectual disability, inguinal hernia syndrome
Fallot complex with intellectual disability and growth delay syndrome
Cortical blindness, intellectual disability, polydactyly syndrome
Epilepsy, microcephaly, skeletal dysplasia syndrome
Intellectual disability Buenos Aires type
Hypotrichosis and intellectual disability syndrome Lopes type
MEDNIK syndrome
Lowry MacLean syndrome
Wilson Turner syndrome
Atkin Flaitz syndrome
X-linked intellectual disability Schimke type
Osteopenia, intellectual disability, sparse hair syndrome
X-linked intellectual disability with seizure and psoriasis syndrome
X-linked intellectual disability Miles Carpenter type
X-linked intellectual disability Brooks type
Okamoto syndrome
MEHMO syndrome
Holmes Gang syndrome
Alport syndrome, intellectual disability, midface hypoplasia, elliptocytosis syndrome
Spastic paraplegia, glaucoma, intellectual disability syndrome
Bullous dystrophy macular type
Facial dysmorphism, macrocephaly, myopia, Dandy-Walker malformation syndrome
Autosomal recessive limb girdle muscular dystrophy type 2K
C syndrome

X-linked intellectual disability and epilepsy with progressive joint contracture and facial dysmorphism syndro
Encephalopathy, intracerebral calcification, retinal degeneration syndrome
Megalocornea with intellectual disability syndrome
Filippi syndrome
Renier Gabreels Jasper syndrome
X-linked intellectual disability Zorick type
Goldblatt Wallis syndrome
X-linked intellectual disability Armfield type
Preaxial polydactyly, colobomata, intellectual disability syndrome
Microcephaly, seizure, intellectual disability, heart disease syndrome
Ichthyosis, intellectual disability, dwarfism, renal impairment syndrome
Juberg Marsidi syndrome
Pallister W syndrome
Temtamy syndrome
X-linked intellectual disability Stocco Dos Santos type
X-linked intellectual disability with plagiocephaly syndrome
Prieto Badia Mulas syndrome
Isodicentric chromosome 15 syndrome
Severe intellectual disability, epilepsy, anal anomaly, distal phalangeal hypoplasia syndrome
MOMO syndrome
Ramos Arroyo syndrome
Epilepsy telangiectasia syndrome
Pseudoprogeria syndrome
Infantile choroidocerebral calcification syndrome
Van den Bosch syndrome
Scholte syndrome
Congenital hypoplasia of ulna and intellectual disability syndrome
X-linked intellectual disability with cerebellar hypoplasia syndrome
5p partial monosomy syndrome
Faciocardiorenal syndrome
Retinitis pigmentosa, intellectual disability, deafness, hypogenitalism syndrome
Coloboma, congenital heart disease, ichthyosiform dermatosis, intellectual disability ear anomaly syndrome
Seizures and intellectual disability due to hydroxylysinuria
X-linked spasticity, intellectual disability, epilepsy syndrome
Stimmler syndrome
X-linked intellectual disability Hedera type
Dentinogenesis imperfecta, short stature, hearing loss, intellectual disability syndrome
Microcephalus, glomerulonephritis, marfanoid habitus syndrome
X-linked intellectual disability Pai type
Chudley Lowry Hoar syndrome
Kleefstra syndrome
Pettigrew syndrome
Disorder of sex development with intellectual disability syndrome
Mild learning disability
Weaver Williams syndrome
Neurofaciodigitorenal syndrome
Deafness and intellectual disability Martin Probst type syndrome
Intellectual disability with cataract and kyphosis syndrome
Short stature, unique facies, enamel hypoplasia, progressive joint stiffness, high-pitched voice syndrome
Alopecia and intellectual disability with hypergonadotropic hypogonadism syndrome

Hair defect with photosensitivity and intellectual disability syndrome
Oculocerebrocutaneous syndrome
Osteogenesis imperfecta, retinopathy, seizures, intellectual disability syndrome
Microcephalus with brachydactyly and kyphoscoliosis syndrome
Aniridia and intellectual disability syndrome
Ectodermal dysplasia with blindness syndrome
Acrodysostosis
Learning disability
Radioulnar synostosis with microcephaly and scoliosis syndrome
Skeletal dysplasia with intellectual disability syndrome
Oro-facial digital syndrome type 11
Agenesis of corpus callosum, intellectual disability, coloboma, micrognathia syndrome
Neuronal ceroid lipofuscinosis 8
Trisomy 10p
Achalasia microcephaly syndrome
Spondyloepiphyseal dysplasia, craniosynostosis, cleft palate, cataract and intellectual disability syndrome
Intellectual disability, developmental delay, contracture syndrome
Complete trisomy 18 syndrome
On learning disability register
Intellectual disability, epilepsy, bulbous nose syndrome
Intellectual disability, craniofacial dysmorphism, hypogonadism, diabetes mellitus syndrome
Oro-facial digital syndrome type 9
Congenital cataract with hypertrichosis and intellectual disability syndrome
Osteopenia, myopia, hearing loss, intellectual disability, facial dysmorphism syndrome
Arachnodactyly with abnormal ossification and intellectual disability syndrome
Skeletal dysplasia with epilepsy and short stature syndrome
Ataxia with deafness and intellectual disability syndrome
Lipodystrophy, intellectual disability, deafness syndrome
Cystic fibrosis with gastritis and megaloblastic anaemia syndrome
Microcephalus cardiomyopathy syndrome
MECP2 duplication syndrome
Renpenning syndrome
Alopecia, contracture, dwarfism, intellectual disability syndrome
Spastic paraplegia, intellectual disability, palmoplantar hyperkeratosis syndrome
Trisomy 21- mitotic nondisjunction mosaicism
4p partial monosomy syndrome
Trisomy 21- meiotic nondisjunction
Intellectual disability, hypoplastic corpus callosum, preauricular tag syndrome
Complete trisomy 13 syndrome
Oculopalatocerebral syndrome
Short stature with webbed neck and congenital heart disease syndrome
Progressive encephalopathy with oedema, hypsarrhythmia and optic atrophy syndrome
Male hypergonadotropic hypogonadism, intellectual disability, skeletal anomaly syndrome
Complete trisomy 21 syndrome
Chromosome 2q37 deletion syndrome
Agammaglobulinaemia, microcephaly, craniosynostosis, severe dermatitis syndrome
Cerebro-facio-thoracic dysplasia
Microcephalus and intellectual disability with phalangeal and neurological anomaly syndrome
Contracture with ectodermal dysplasia and orofacial cleft syndrome
Seizure, sensorineural deafness, ataxia, intellectual disability, electrolyte imbalance syndrome

Cutis laxa-corneal clouding-oligophrenia syndrome
Macrocephaly, short stature, paraplegia syndrome
19q13.11 microdeletion syndrome
Moderate intellectual development disorder without significant impairment of behaviour
Profound intellectual development disorder with minimal impairment of behaviour
Macrocephaly and developmental delay syndrome
Intellectual disability, myopathy, short stature, endocrine defect syndrome
Mild intellectual development disorder with impairment of behaviour
Profound intellectual development disorder without impairment of behaviour
Severe intellectual development disorder with impairment of behaviour
Pachygyria, intellectual disability, epilepsy syndrome
Severe intellectual development disorder with minimal impairment of behaviour
Moderate intellectual development disorder with significant impairment of behaviour
Diencephalic mesencephalic junction dysplasia
Malan overgrowth syndrome
Intellectual disability, brachydactyly, Pierre Robin syndrome
Intellectual disability Birk-Barel type
Intellectual disability, alacrima, achalasia syndrome
Moderate intellectual development disorder with impairment of behaviour
Intellectual disability, spasticity, ectrodactyly syndrome
Intellectual development disorder without significant impairment of behaviour
Intellectual development disorder with significant impairment of behaviour
Severe intellectual development disorder with significant impairment of behaviour
Profound learning disability
Intellectual disability, congenital heart disease, blepharophimosis, blepharoptosis and hypoplastic teeth
Ichthyosis, alopecia, eclabion, ectropion, intellectual disability syndrome
Craniofaciofrontodigital syndrome
X-linked intellectual disability with marfanoid habitus
X-linked intellectual disability, hypogonadism, ichthyosis, obesity, short stature syndrome
Alpha-thalassaemia intellectual disability syndrome linked to chromosome 16
Intellectual disability Wolff type
HIVEP2-related intellectual disability
Brachydactyly, mesomelia, intellectual disability, heart defect syndrome
Moderate learning disability
Grubben, De Cock, Borghgraef syndrome
Mild learning disability
Profound intellectual development disorder with significant impairment of behaviour
Profound intellectual development disorder with impairment of behaviour
SCN8A-related epilepsy with encephalopathy
Severe intellectual development disorder without significant impairment of behaviour
Severe learning disability
Mild intellectual development disorder with minimal impairment of behaviour
Craniodigital syndrome and intellectual disability syndrome
Aortic arch anomaly, facial dysmorphism, intellectual disability syndrome
Cryptorchidism, arachnodactyly, intellectual disability syndrome
Charcot-Marie-Tooth disease, deafness, intellectual disability syndrome
Hyperphosphatasaemia with intellectual disability
Mild intellectual development disorder without significant impairment of behaviour
Intellectual disability due to nutritional deficiency
Ohdo syndrome, Maat-Kievit-Brunner type

Focal epilepsy, intellectual disability, cerebro-cerebellar malformation syndrome
Hypotonia, speech impairment, severe cognitive delay syndrome
Mild intellectual development disorder with significant impairment of behaviour
Intellectual disability, polydactyly, uncombable hair syndrome
Epiphyseal dysplasia, hearing loss, dysmorphism syndrome
Intellectual disability, obesity, brain malformation, facial dysmorphism syndrome
Moderate intellectual development disorder with minimal impairment of behaviour
Agenesis of corpus callosum and abnormal genitalia syndrome
PPP2R5D-related intellectual disability
Coffin-Siris syndrome
Oro-facial digital syndrome type 14
Intellectual development disorder with impairment of behaviour
Intellectual development disorder with minimal impairment of behaviour
Wiedemann Steiner syndrome
Rett's disorder
De Lange syndrome
Bardet-Biedl syndrome
Dubowitz's syndrome
Mucopolysaccharidosis, MPS-I-H
Kohlschutter's syndrome
Cohen syndrome
Nijmegen breakage syndrome-like disorder
Borjeson-Forssman-Lehmann syndrome
Cross syndrome
Seckel syndrome
Lowe syndrome
Prader-Willi syndrome
Angelman syndrome
Coffin-Lowry syndrome
Partial trisomy 13 in Patau's syndrome
Partial trisomy 21 in Down's syndrome
Molybdenum cofactor deficiency complementation group B
Hepatic fibrosis, renal cyst, intellectual disability syndrome
Significant learning disability
Maternal 15q11q13 deletion
Autosomal recessive frontotemporal pachygyria
Cyclin-dependent kinase-like 5 deficiency
10q22.3q23.3 microdeletion syndrome
Alpha-N-acetylgalactosaminidase deficiency type 1
Bilateral frontoparietal polymicrogyria
Woodhouse Sakati syndrome
Severe intellectual disability and progressive spastic paraplegia
Primary microcephaly, mild intellectual disability, young-onset diabetes syndrome
Rhizomelic syndrome Urbach type
49,XXXYY syndrome
Ataxia, photosensitivity, short stature syndrome
Ring chromosome 12 syndrome
9q34 deletion syndrome
Distal trisomy 18q
Congenital muscular dystrophy with intellectual disability

Alopecia, epilepsy, intellectual disability syndrome Moynahan type
Microcephaly with simplified gyral pattern
X-linked intellectual disability due to GRIA3 mutations
Distal Xq28 microduplication syndrome
Severe feeding difficulties, failure to thrive, microcephaly due to ASXL3 deficiency syndrome
Severe microbrachycephaly, intellectual disability, athetoid cerebral palsy syndrome
Polyneuropathy, intellectual disability, acromicria, premature menopause syndrome
Kagami Ogata syndrome
Pseudoleprechaunism syndrome Patterson type
Intellectual disability with strabismus syndrome
Infantile spasms, psychomotor retardation, progressive brain atrophy, basal ganglia disease syndrome
Pitt Hopkins-like syndrome
Intellectual disability, coarse face, macrocephaly, cerebellar hypotrophy syndrome
Polymicrogyria with optic nerve hypoplasia
Severe intellectual disability, progressive spastic diplegia syndrome
Intellectual disability, seizures, macrocephaly, obesity syndrome
12q15 deletion syndrome
Hypogonadotropic hypogonadism, severe microcephaly, sensorineural hearing loss, dysmorphism syndrome
Down syndrome co-occurrent with leukemoid reaction associated transient neonatal pustulosis
Severe intellectual disability, short stature, behavioural abnormalities, facial dysmorphism syndrome
Molybdenum cofactor deficiency complementation group C
Mowat-Wilson syndrome due to monosomy 2q22
Developmental delay, facial dysmorphism syndrome due to MED13L deficiency
Tetrasomy 11q24.1
Rare non-syndromic intellectual disability
3q27.3 microdeletion syndrome
Progressive encephalopathy with oedema, hypsarrhythmia, and optic atrophy-like syndrome
X-linked complicated corpus callosum dysgenesis
CK syndrome
AHDC1-related intellectual disability, obstructive sleep apnoea, mild dysmorphism syndrome
Cockayne syndrome type 2
3p25.3 deletion syndrome
White matter hypoplasia, corpus callosum agenesis, intellectual disability syndrome
Autism spectrum disorder due to AUTS2 deficiency
Autism spectrum disorder due to AUTS2 deficiency
Ophthalmoplegia, intellectual disability, lingua scrotalis syndrome
Richieri Costa-da Silva syndrome
Congenital muscular dystrophy with intellectual disability and severe epilepsy
Developmental and speech delay due to SOX5 deficiency
Alpha-N-acetylgalactosaminidase deficiency type 3
MASA syndrome
Monosomy 9p
Intellectual disability, craniofacial dysmorphism, cryptorchidism syndrome
Mosaic 1q duplication
Autosomal recessive intellectual disability, motor dysfunction, multiple joint contracture syndrome
Alpha-N-acetylgalactosaminidase deficiency type 2
Bilateral frontal polymicrogyria
Autosomal recessive cerebellar ataxia, epilepsy, intellectual disability syndrome due to TUD deficiency
Microcephalic primordial dwarfism Alazami type
Intellectual disability, facial dysmorphism syndrome due to SETD5 haploinsufficiency

Bilateral megalencephaly
Intellectual disability, seizures, hypotonia, ophthalmologic, skeletal anomalies syndrome
Distal monosomy 19p13.3
Microlissencephaly
Monosomy 13q14 syndrome
Acrocardiofacial syndrome
White Sutton syndrome
Cockayne syndrome type 1

ome
